# Two Flavones as Markers for the Distinction of Natural Acacia Honey Based on the UHPLC‐QE‐MS Method and Research on Their Potential Immunomodulatory Activity

**DOI:** 10.1002/fsn3.72021

**Published:** 2026-06-17

**Authors:** Bingkang Wang, Min Hua, Yuecheng Liu, Meng Li, Ning Zhang, Yi Wu, Qi Gao, Hongfu Sun, Yanpeng Dai, Meng Zhou, Qian Zhou

**Affiliations:** ^1^ College of Pharmacy Shandong University of Traditional Chinese Medicine Jinan China; ^2^ College of Pharmacy Yantai University Yantai China; ^3^ Shandong Academy of Chinese Medicine Jinan China; ^4^ Shandong Modern Research and Development Engineering Center of Traditional Chinese Medicine Aromatherapy Jinan China; ^5^ College of Veterinary Medicine Yunnan Agricultural University Kunming China; ^6^ The Key Unit for Research of Technique and Principle of Honey‐Processing and Carbonizing of SATCM Jinan China; ^7^ Department of Pharmacy, the Second Qilu Hospital, Cheeloo College of Medicine Shandong University Jinan China

**Keywords:** acacia honey, immune regulation, quality evaluation, quality markers, SPE‐UPLC‐Q‐Exactive Orbitrap MS

## Abstract

Honey is a natural sweet substance produced by bees from nectar and plant secretions, which is abundant in nutrients and exhibits excellent biological activities. However, owing to deficiencies in the quality control index system, prominent issues such as honey adulteration and improper processing are prevalent in the current market, resulting in a severe decline in honey quality. Therefore, this study focuses on acacia honey, one of the four major honey varieties in China, by screening its characteristic components and analyzing the correlation between these components and immunomodulatory activity, aiming to provide targeted theoretical basis and indicator support for resolving the aforementioned practical issues. The results demonstrated that natural acacia honey (NAH) had significant advantages over commercial acacia honey (CAH) in key quality indices (total flavonoids, total phenols, proline content, amylase activity, etc.) and quality consistency, with significantly higher immunomodulatory activity. Combined with machine learning and chemometric analyses, hesperetin and pinocembrin were confirmed as the characteristic components of NAH from the 50 components identified via ultra‐performance liquid chromatography‐quadrupole‐electrostatic field orbitrap mass spectrometry (UPLC‐QE‐MS) analysis. In vitro experiments demonstrated that both components at 50 μM effectively promoted mouse splenic lymphocyte proliferation, reduced NO and TNF‐α secretion in LPS‐induced RAW264.7 macrophages, and showed no obvious cytotoxicity, indicating their potential as characteristic markers for evaluating NAH quality and functional activity. Further network pharmacology and molecular docking studies indicated that hesperetin and pinocembrin could mediate immunomodulatory effects by regulating 10 core targets including MMP9, MMP2, BCL2, and SRC, as well as the PI3K‐AKT pathway, with the markers showing the strongest binding activity toward MMP2. This study established a characteristic marker identification system for NAH, and provided scientific indicator support for formulating acacia honey quality standards and improvement of processing technologies.

## Graphical abstract

This study systematically compared the quality characteristics and immunomodulatory activities of natural and commercial honey. A comprehensive strategy combining physicochemical determination, SPE‐UPLC‐Q‐Exactive Orbitrap MS‐based metabolomics, and machine learning‐assisted chemometrics was established to screen differential biomarkers. Hesperetin and pinocembrin were identified as the core markers distinguishing the two types of honey. Network pharmacology and in vitro immune assays were applied to explore the immunomodulatory mechanism. Molecular docking and molecular dynamics simulation further validated that the PI3K‐AKT signaling pathway and core target MMP2 serve as the key molecular basis for the immunomodulatory effects of natural honey. This work provides scientific support for honey quality identification and functional product development.

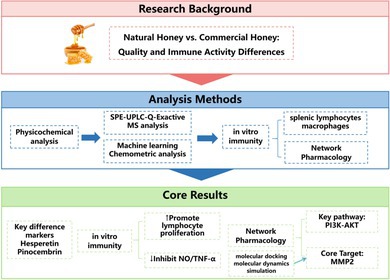

**Abbreviations**
AUC: area under the curve
CA: classification accuracy
Con A: Concanavalin A
F1: F1 score
FEL: free energy landscape
GB‐ANOVA: gradient boosting‐analysis of variance
GO: gene ontology
HCA: hierarchical cluster analysis
KEGG: Kyoto encyclopedia of genes and genomes
LPS: lipopolysaccharide
MCC: matthews correlation coefficient
ML: machine learning
MMP2: matrix metalloproteinase 2
NN‐ANOVA: neural network‐analysis of variance
NO: nitric oxide
OPLS‐DA: orthogonal partial least squares discriminant analysis
PCA: principal component analysis
PI3K‐AKT: Phosphoinositide 3‐Kinase/Akt Serine/Threonine Kinase
PPI: protein–protein interaction
Prec: precision
RF‐X^2^
: random forest‐chi‐squared test
Rg: radius of gyration
RMSD: root‐mean‐square deviation
RMSF: root mean square fluctuation
SASA: solvent‐accessible surface area
SD: standard deviation
SVM‐IG: support vector machine‐information gain
TNF‐α: tumor necrosis factor‐alpha
TREE‐ReliefF: decision tree‐reliefF algorithm
UPLC‐QE‐MS: ultra performance liquid chromatography‐ Q exactive‐mass spectrometry
VIP: Variable importance in projection

## 1 Introduction

Honey is a natural sweet substance produced by honeybees from nectar, plant secretions, or honeydew, with the addition of the bees' own specific substances (da Silva et al. 2016). Primarily composed of fructose and glucose, it also contains diverse bioactive metabolites, including proteins, free amino acids, trace minerals, organic acids, and flavonoids (Luca et al. 2025). These bioactive components collectively endow honey with excellent nutritional value and medicinal potential, as well as a broad spectrum of biological activities (Akanda et al. 2024). Owing to its superior nutritional profile relative to conventional synthetic sweeteners (e.g., sucrose, aspartame), honey has garnered high consumer acceptance and is now widely utilized in the food, cosmetics, healthcare, and pharmaceutical industries (Sun 2017).

However, issues such as intentional adulteration and non‐compliant production have severely hindered the healthy development of the global honey industry, including China's sector. A key cause of this problem is the lack of scientific and controllable quality control indicators for honey. Currently, glucose and fructose remain the core indicators, a trait that renders honey highly vulnerable to exploitation by illegal actors during processing, thereby facilitating adulteration practices. Meanwhile, insufficient attention often leads to the loss or degradation of other nutritional and bioactive components in processing. Even acacia honey (AH), one of China's four renowned honeys, is not immune to these issues (Sun 2022).

Honey's endogenous components reflect its natural quality, playing a key role in evaluating nutrition value, assessing product quality and detecting adulteration. Thus, in recent years, analytical methods for honey traceability and authenticity identification based on endogenous components have become a research hotspot in the field of food science (Zhang et al. 2017). Existing studies confirmed that 8‐hydroxyoctanoic acid can effectively distinguish between honey from *Apis cerana* (Chinese honeybee) and *Apis mellifera* (Italian honeybee) (Liu et al. 2023); sebacic acid could flag mature and immature brassica honey (Sun et al. 2021); and unique chemical markers like methyl syringate 4‐O‐β‐D‐gentiobioside and leucidin can identify meluca honey (Lin et al. 2020; Ren et al. 2022). Endogenous markers possess high specificity and stability, and are closely associated with antibacterial and antioxidant bioactivities, and are technically advantageous for easy detection and resistance to adulteration. When used as core honey quality control indicators, they can not only provide a scientific basis for the supervision and quality management of the honey industry, but also guide the reverse screening of honey raw materials and the optimization and upgrading of production processes. However, to date, no systematic research on phytochemical markers associated with the bioactivities of NAH has been reported.

Therefore, this study aims to identify the characteristic bioactive components in NAH as its core objective. First, systematic detection and analysis were performed on key quality parameters, including specific enzyme activities, natural polyphenol contents, and characteristic sugar component ratios, to comprehensively compare and scientifically evaluate the quality discrepancies between NAH and CAH. Subsequently, a multi‐dimensional technical approach integrating machine learning modeling, chemometric analysis, in vitro cell functional validation, and network pharmacology prediction was employed to screen and identify the characteristic components of AH, as well as clarify the intrinsic correlations and potential mechanisms of action between these components and immunomodulatory effects. Collectively, this research can not only provide technical support for the precise screening of high‐quality AH and the optimization of production processes but also lay a solid theoretical foundation for the in‐depth elucidation of the nutritional and health‐promoting benefits of natural honey.

## 2 Materials and Methods

### 2.1 Materials

Ethanol, sodium nitrite, aluminum nitrate, sodium hydroxide, Folin–Ciocalteu reagent, sodium carbonate, ninhydrin, ethylene glycol monomethyl ether, isopropanol, iodine, potassium iodide, soluble starch, sodium acetate, glacial acetic acid (analytically pure), sodium chloride, hydrochloric acid, LC–MS grade acetonitrile, and methanol (Fisher Chemical) from Thermo Fisher Scientific. PBS, 1640 medium, fetal bovine serum, DMEM medium, penicillin, and streptomycin (Gibco). NO and CCK‐8 detection kit (Beyotime Biotechnology). ELISA detection kits (Shanghai Enzyme‐linked Biotechnology). Fructose, glucose, sucrose, lactose, quercetin, gallic acid, and proline standard (purity 98%, Herbpurify). Ultrapure water from the MilliQ system (Millipore Corporation, Billerica, MA, USA) was used throughout the study.

### 2.2 Sample Preparation

Fifteen batches of NAH were collected from a certified single‐flora AH base in Liaocheng, Shandong during the 2024 acacia flowering season (April–May). The botanical origin ( *Robinia pseudoacacia* L.) was verified by field investigation, beekeeping traceability records, and supplier qualification certification, ensuring all samples were authentic and pure AH. All samples were naturally mature, unheated, and unprocessed. Twelve batches of CAH were purchased from the market, all labeled as pure or single‐flora acacia honey with *Robinia pseudoacacia* declared as the sole nectar source. All samples were analyzed in three biological replicates and stored at 4°C under identical dark and sealed conditions. By unifying the declared floral source and storage regime, the comparability between NAH and CAH was well ensured, and the interference from uncontrolled external factors was minimized.

### 2.3 Physicochemical Analysis

#### 2.3.1 Moisture Content

The moisture content of the honey samples was determined by measuring the refractive index of each sample using an Abbe refractometer and the obtained value was substituted into the following formula for calculation: Moisture (%) = 100–[78 + 390.7 (*n*–1.4768)] to calculate the moisture content. In the formula, n represents the actual refractive index of honey samples measured at 40°C (GB 14963‐2011 National Food Safety Standard‐Honey 2012).

#### 2.3.2 pH Value

The acidity of honey was determined using a pH meter. 1 g of honey was dissolved in 7.5 mL of deionized water, vortexed, and subjected to pH measurement at room temperature.

#### 2.3.3 Total Flavonoid Content (TFC)

The TFC in honey was determined using the aluminum nitrate colorimetric method (An et al. 2025). 1 mL of honey (80% w/v in 30% ethanol) was mixed with 0.4 mL of NaNO_2_ solution (50 g/L) and placed for 6 min. Then 0.4 mL of AI (NO_3_)_3_ solution (100 g/L) was added and shaken for 6 min. 4 mL of NaOH solution (40 g/L) was added and diluted to 10 mL with 30% ethanol, mix well and place for 15 min. The absorbance at 510 nm was measured using a multifunctional microplate reader (Bio‐Red Laboratories Inc.), and the standard curve was obtained using quercetin (0–400 μg/mL) as the standard. TFC content was expressed as quercetin equivalent (mg QE/100 g).

#### 2.3.4 Total Phenol Content (TPC)

The content of TPC in honey was determined by the Folin–Ciocalteu colorimetric method (Lawag et al. 2023). 1 mL honey (20% w/v in water) with 1 mL FolinCiocalteu reagent were mixed and let stand for 5 min. After that, 5.0 mL sodium carbonate solution (1 mol/L) was added and diluted to 10 mL with deionized water and incubated for 1 h at room temperature in the dark. The absorbance at 760 nm was measured using a multifunctional microplate reader (Bio‐Rad Laboratories Inc.), and the standard curve was obtained using gallic acid (0–400 μg/mL) as the standard. TPC content was expressed as gallic acid equivalent (mg QE/100 g).

#### 2.3.5 Proline Content

The proline content was modified according to the determination method of Damto et al. (2024). 1 mL honey (10% w/v in water) was mixed with 250 μL formic acid and 1 mL ninhydrin‐ethylene glycol monomethyl ether solution (3% w/v in ethylene glycol monomethyl ether). The mixture was heated in boiling water for 15 min, and then placed in a water bath at 70°C for 15 min. 5 mL isopropanol aqueous solution was added (v/v, 1:1) and stand at room temperature for 5 min. The absorbance was measured at 510 nm. The standard curve was obtained using proline (0–400 μg/mL) as the standard. Proline content was expressed as mg/100 g.

#### 2.3.6 Diastase

The diastase activity was determined according to the Chinese national standard GB 14963‐2011 (National Food Safety Standard‐Honey 2012), which is the official standard method for honey quality evaluation in China. The procedure was as follows:

Preparation of starch solution: 2 g of soluble starch was dissolved in 90 mL of boiling water, boiled for 3 min, cooled to room temperature, and diluted to 100 mL with water.

Sample preparation: 5 g of honey (accurate to 0.01 g) was mixed with 15 mL of water, 2.5 mL of acetate buffer (pH 5.3, 1.59 mol/L), and 1.5 mL of 0.5 mol/L NaCl solution, then diluted to 25 mL.

Dilution coefficient determination: 5 mL starch solution and 10 mL water were pre‐incubated at 40°C for 15 min. 1 mL of the mixture was added to 10 mL iodine solution and diluted with water to an absorbance of 0.760 ± 0.02 at 660 nm. The volume of water added was defined as the dilution coefficient (D).

Determination: 5 mL of starch solution and 10 mL of sample solution were pre‐incubated at 40°C for 15 min. The reaction was initiated by mixing the two solutions. At 5 min intervals, 1 mL reaction mixture was added to 10 mL iodine solution, diluted with D mL water, and the absorbance was measured at 660 nm. The reaction was stopped when the absorbance was below 0.235.

Calculation: Diastase activity (mL/(g·h)) = 300/*t*, where *t* is the time (min) required for the absorbance to drop below 0.235.

### 2.4 UPLC‐Q‐Exactive Orbitrap‐MS Analysis

#### 2.4.1 Sample Preparation

Each honey sample (10 g) was mixed with acidified water (50 mL), which was adjusted to pH 2 with calibrated hydrochloric acid. The sample was centrifuged at 10,000 r/min for 5 min at 4°C to remove the solid particles. The supernatant sample was loaded onto HLB column (3 mL methanol, 3 mL ultrapure water activation). Then, the cartridge was rinsed with 4 mL of acidified ultrapure water (pH 2) to remove saccharides and other polar compounds. Phenolic and flavonoid compounds absorbed on the cartridge were eluted with 5 mL of 2% formic acid‐methanol solution. The methanol solution was evaporated to dryness by nitrogen blowing, and the residue was reconstituted in 2 mL of methanol. All solution samples were filtered through a 0.22 μm syringe filter from Waters Corporation prior to UPLC injection (Li et al. 2023).

#### 2.4.2 UPLC‐Q‐Exactive Orbitrap‐MS Instrumentation

The extract was analyzed using a Thermo Fisher UltiMate 3000 UPLC (Thermo Fisher Scientific, Waltham, Massachusetts, USA). Separation was achieved on a Waters ACQUITY UPLC HSS T3 C18 column (2.1 × 100 mm, 1.8 μm) using a mobile phase that consisted of 0.1% formic acid in water (A) and acetonitrile (B) with the following gradient program (v/v): 0–2 min, 98% B; 2–3 min, 95%–83% B; 3–12 min, 83% B; 12–14 min, 83%–71% B; 14–19 min, 71% B; 19–31 min, 71%–5% B; 31–31.1 min, 5%–98% B. The flow rate was 0.3 mL/min, the column temperature was set at 40°C, and the injection volume was 3 μL.

Mass spectrometry was performed on a Thermo Fisher Q‐Exactive Orbitrap MS system (Thermo Fisher Scientific, Waltham, MA, USA). An electrospray ionization (ESI) ion source was adopted in negative ion mode. For negative ESI analysis, the source parameters were set as follows: capillary voltage at 3500 V, capillary temperature at 350°C, nitrogen (N_2_) as sheath gas at 35.0 L/min, nitrogen (N_2_) as auxiliary gas at 10.0 L/min, fragmentation voltage at 60 V, and collision energies of 30.0, 50.0, and 70.0 eV. The full‐scan mass range was 100–1500 Da.

### 2.5 Machine Learning

Machine learning algorithms, including NN (hidden layer size = 25, activation = ReLU, solver = Adam), RF (number of trees = 4, max depth = 5), SVM (kernel = RBF, C = 1.2), and KNN (number of neighbors = 4, metric = Euclidean, weights = uniform), were utilized to classify the different types of honey samples. Key parameters of each model were optimized via 10‐fold cross validation to reduce bias. The validation process involving random sampling was performed 10 times, with a training‐to‐testing set ratio of 8:2. The neural network uses an early stopping strategy (maximum number of iterations = 100), the random forest limits the maximum depth (the maximum depth of a single decision tree is capped at 5), and the support vector machine uses regularization (regularization parameter C = 1). The Information Gain (IG), Feature Weight (Relief F), χ^2^ (chi‐square test), and ANOVA (analysis of variance) were utilized to rank the importance of feature values. The machine learning and feature selection were combined to distinguish subsets of features that maximize model performance. This process is designed to evaluate the prediction accuracy of the model for different feature subsets, which can determine the most efficient feature subset.

### 2.6 Effect of Honeys and Chemical Markers on the Proliferation of Spleen Lymphocytes

All animal studies were approved by the Animal Ethics Committee of Shandong Provincial Institute of Chinese Medicine (Jinan, China; Approval No.: SDZYY20230317004) and conducted in accordance with guidelines established by the European Community (EEC Directive 1986; 86/609/EEC). Male Balb/c mice aged 8 weeks (20 ± 2 g) were purchased from Shandong Pengyue Experimental Animal Technology Co. Ltd. (Approval No.: SYXK (Lu) 2022‐0006, Jinan, China) and acclimatized under sterile conditions to controlled humidity (55%–60%) and temperature (22°C–24°C), as well as a 12:12‐h light cycle.

BALB/c mice were sacrificed by cervical dislocation, and the spleen was ground on a 200‐mesh sieve to collect spleen cells. Red blood cells were lysed with red blood cell lysate. After centrifugation at 4°C, 450 g for 10 min, the supernatant was discarded, and the cells were resuspended in RPMI 1640 medium to obtain spleen lymphocytes.

The cell concentration was adjusted to 4 × 10^6^ cells/mL. 96‐well cell culture plate, 100 μL cell suspension was added to each well, and 100 μL honey sample was added to the drug group. Lipopolysaccharide (LPS) ‐induced cells or concanavalin A (Con A) ‐induced group was added with 80 μL of sample and 20 μL of LPS or Con A dissolved in medium (The final concentrations of samples were 7.5, 12.5, 25 mg/mL for NAH, 0.5, 2.5, 7.5 mg/mL for commercially available honey and 10 μg/mL for LPS and Con A). Honey control group, Con A control group and LPS control group were set up. Each group has 4 wells. The culture plates were placed in a 5% CO_2_ incubator at 37°C and saturated humidity for 24 h. At the end of culture, 20 μL CCK‐8 was added to each well and incubated for 2 h. The absorbance (A) was detected at 450 nm and the cell proliferation rate was calculated. Proliferation rate (%) = (administration group A value/control group A value) × 100% (Ghramh et al. 2021).

### 2.7 Inflammation Activity Assay of Markers

RAW264.7 cells in logarithmic growth phase were taken and the concentration was adjusted to 1 × 10^5^ cells/mL. Inoculated in 96‐well plates, 100 μL per well, control group and drug group were set up. Each group was set up with 3 replicates. After 24 h incubation in 37°C, 5% CO_2_ incubator, different concentrations of chemical markers (hesperetin and pinocembrin) were added to stimulate for 24 h. CCK‐8 solution was added and incubated for 1.5 h. The absorbance of each hole was measured by a microplate reader at a wavelength of 450 nm, and the cell survival rate was calculated. Survival rate (%) = A (administration group)/A (control group) × 100%.

Cells in logarithmic growth phase were taken and the concentration was adjusted to 1.5 × 10^5^ cells/mL. Inoculated in 6‐well plates, 2 mL per well, control group and drug group were set up. The administration group was added with complete medium containing different concentrations of chemical markers, and the other groups were added with 2 mL complete medium, and then cultured for 24 h. A certain amount of LPS was added to the model group and the administration group to a final concentration of 1 μg/mL. After 24 h of stimulation, the supernatant was taken to determine the production of NO and TNF‐α according to the Griess and ELISA kit methods (Biluca et al. 2020).

### 2.8 Network Pharmacology Analysis

Multiple databases were combined to obtain the targets of two chemical markers (hesperetin and pinocembrin), including TCMSP (https://www.tcmsp‐e.com/), HERB (http://herb.ac.cn/), Swiss Target Prediction (http://swisstargetprediction.ch/), which were further matched to their corresponding standardized gene names using the Universal Protein Resource database (https://www.uniprot.org), with species being set as “ *Homo sapiens* .”

The potential targets that were also linked to the keyword “inflammation” and “enhance immunity” in the databases GeneCards (https://www.genecards.org) or Online Mendelian Inheritance in Man (OMIM, https://www.omim.org) were identified using the Venny 2.1.0 tool (http://bioinfogp.cnb.csic.es/ tools/venny/index.html) and retained for further analysis. Protein–protein interaction (PPI) networks involving inflammation or enhance immunity targets were explored using Cytoscape 3.9.1 software, and their enrichment in Gene Ontology (GO) terms and Kyoto Encyclopedia of Genes and Genomes (KEGG) pathways was explored using the DAVID database (https://david.ncifcrf.gov). The inflammation or enhance immunity targets, two chemical markers, and the top 20 KEGG pathways were integrated into a “component‐target‐pathway‐disease” network using Cytoscape 3.9.1 software.

### 2.9 Molecular Docking

This study conducted the molecular docking of two compounds with key targets (MMP2). We obtained the 2D file from PubChem (https://pubchem. ncbi.nlm.nih.gov/), and Chem3D 22.0.0 was used to optimize the structure. Moreover, we collected the X‐ray crystal structures for the receptor proteins based on the PDB database (https://www.pdburcsb.org/). CB‐Dock2 (https://cadd.labshare.cn/cb‐dock2/) was used to process the structures and conduct molecular docking simulations. Ultimately, visualization of molecular docking results of the best conformations was performed with Discovery Studio 2019.

### 2.10 Molecular Dynamics Simulation

Molecular dynamics (MD) simulations were performed using GROMACS 2022. The protein topology was generated with the AMBER14SB force field using the pdb2gmx module. Ligand parameters were obtained using the GAFF2 force field via the sobtop_1.0 (dev3.1) program, and atomic charges were assigned using the RESP method.

The protein‐ligand complex was solvated in a cubic box with a 1.0 nm buffer distance using the TIP3P water model. To maintain electroneutrality and mimic physiological conditions, Na^+^ and Cl^−^ ions were added to achieve a final concentration of 0.15 M using the gmx genion tool. Long‐range electrostatic interactions were treated using the Particle Mesh Ewald (PME) method with a cutoff distance of 1.0 nm. All covalent bonds were constrained using the LINCS algorithm. Prior to MD simulation, energy minimization was conducted using the steepest descent method (3000 steps), followed by the conjugate gradient method (2000 steps). The system was then equilibrated under standard conditions. MD simulations were carried out under the NPT ensemble at 310 K and 1 bar, maintained by the Nosé‐Hoover thermostat and Parrinello‐Rahman barostat, respectively. The simulation time was set to 100 ns with a time step of 2 fs.

Trajectory analyses were performed using GROMACS built‐in tools, including RMSD, RMSF, hydrogen bonds, radius of gyration (Rg), and solvent‐accessible surface area (SASA), to evaluate the structural stability and dynamic behavior of the system.

### 2.11 Statistical Analysis

The determination of physical and chemical indicators was repeated three times, and statistical analysis was performed using SPSS27. The data were expressed as mean ± standard deviation (SD). Mass spectrometry data were collected using Xcalibur software. The mass spectrometry data were corrected by R3.2.5 and imported into MATLAB for artificial neural network (clustering) analysis. The data were imported into SIMCA‐P software (v13.0, Umetrics, Umeå, Sweden) for principal component analysis (PCA), hierarchical cluster analysis (HCA), and orthogonal partial least squares discriminant analysis (OPLS‐DA). Statistical analyses were performed using Student's *t*‐tests or one‐way ANOVA using GraphPad Prism 9. Prior to statistical analysis, normality and homogeneity of variance were examined to verify that all data satisfied the assumptions of parametric tests. For multiple comparisons, the Bonferroni correction was applied to control type I error. The statistical significance was *p < 0.05, **p < 0.01, ***p < 0.001.

## 3 Results and Discussion

### 3.1 Physicochemical Characteristics of AH

The physicochemical indices of AH samples were summarized in Table 1. Among these indices, moisture content is a critical parameter for assessing honey quality, as it serves as a key indicator for evaluating honey maturity, viscosity, and storage stability (Živkov Baloš et al. 2023). For all AH samples analyzed in this study, the moisture content ranged from 16.22% to 18.56%. This range fully complied with the moisture content limit (≤ 20%) specified in relevant Chinese national standards for honey (e.g., GB 14963‐2024 National Standard for Honey), indicating that the tested AH samples met the basic quality requirements for commercial circulation.

**TABLE 1:** Physicochemical characteristics of AH.

	Moisture (%)	Reducing sugar (%)	Sucrose (%)	pH	TFC (mg/100 g)	TPC (mg/100 g)	Proline content (mg/100 g)	Amylase
N1	18.0 ± 0.10^efg^	62.72 ± 0.02^efg^	0.19 ± 0.01	4.30 ± 0.02^lmn^	18.533 ± 1.806^def^	70.00 ± 6.54^fgh^	80.14 ± 0.34^bc^	28.72 ± 0.32^a^
N2	17.9 ± 0.06^efg^	63.89 ± 0.01^def^	0.19 ± 0.03	4.24 ± 0.02^o^	18.460 ± 1.365^def^	67.91 ± 2.43^fgh^	80.31 ± 0.48^bc^	27.11 ± 0.54^b^
N3	18.0 ± 0.12^ef^	62.61 ± 0.02^efg^	0.19 ± 0.01	4.28 ± 0.02^mn^	19.112 ± 0.070^cdef^	68.04 ± 2.25^fgh^	77.17 ± 2.39^cd^	24.91 ± 1.13^cd^
N4	18.0 ± 0.12^efg^	65.28 ± 0.02^bc^	0.21 ± 0.03	4.27 ± 0.01^n^	18.340 ± 0.870^def^	68.01 ± 2.24^fgh^	79.90 ± 0.78^bc^	25.77 ± 0.03^c^
N5	17.9 ± 0.06^efg^	63.96 ± 0.02^def^	0.21 ± 0.05	4.22 ± 0.01^o^	19.321 ± 0.885^cdef^	69.05 ± 2.28^fgh^	77.58 ± 3.11^cd^	25.58 ± 0.36^cd^
N6	18.1 ± 0.06^e^	64.03 ± 0.01^cde^	0.22 ± 0.02	4.17 ± 0.03^p^	19.913 ± 1.503^bcd^	68.43 ± 2.81^fgh^	78.69 ± 2.27^bc^	27.26 ± 0.79^b^
N7	18.0 ± 0.10^efg^	64.84 ± 0.02^bcd^	0.18 ± 0.03	4.30 ± 0.01^lm^	18.052 ± 0.072^ef^	65.38 ± 2.31^h^	75.64 ± 4.37^cd^	27.62 ± 1.15^b^
N8	18.1 ± 0.06^e^	64.52 ± 0.02^bcd^	0.21 ± 0.02	4.29 ± 0.01^lmn^	18.789 ± 0.101^cdef^	66.87 ± 3.19^gh^	79.24 ± 0.79^bc^	16.58 ± 0.50^e^
N9	17.9 ± 0.06^efg^	64.99 ± 0.01^bcd^	0.23 ± 0.03	4.28 ± 0.02^mn^	20.357 ± 1.684^bc^	68.43 ± 2.31^fgh^	80.29 ± 1.76^bc^	14.75 ± 0.71^f^
N10	18.0 ± 0.12^ef^	64.34 ± 0.01^cde^	0.2 ± 0.04	4.32 ± 0.02^l^	22.312 ± 1.405^a^	67.39 ± 2.62^gh^	78.10 ± 1.96^bcd^	17.19 ± 0.69^e^
N11	17.9 ± 0.06^efg^	66.26 ± 0.02^b^	0.22 ± 0.04	4.45 ± 0.02^ij^	21.134 ± 1.535^ab^	71.91 ± 2.25^efgh^	77.24 ± 4.27^cd^	14.32 ± 0.11^f^
N12	18.0 ± 0.12^efg^	64.82 ± 0.01^bc^	0.19 ± 0.02	4.44 ± 0.01^ij^	18.693 ± 0.881^cdef^	69.57 ± 3.95^fgh^	79.82 ± 2.32^bc^	24.60 ± 0.56^d^
N13	18.0 ± 0.10^efg^	64.94 ± 0.01^bc^	0.2 ± 0.02	4.42 ± 0.01^jk^	19.761 ± 0.438^bcde^	64.56 ± 2.24^h^	73.02 ± 6.75^de^	24.61 ± 0.27^d^
N14	17.8 ± 0.06^g^	65.08 ± 0.01^bc^	0.19 ± 0.01	4.43 ± 0.02^ijk^	22.627 ± 1.039^a^	77.24 ± 3.19^e^	74.57 ± 3.74^cd^	24.81 ± 0.73^cd^
N15	17.9 ± 0.06^fg^	65.00 ± 0.01^bc^	0.21 ± 0.04	4.41 ± 0.01^k^	17.873 ± 0.912^f^	65.23 ± 2.40^h^	76.62 ± 0.26^cd^	24.56 ± 0.64^d^
C1	18.4 ± 0.10^d^	68.61 ± 0.01^a^	0.18 ± 0.01	5.62 ± 0.02^b^	3.543 ± 0.158^k^	53.60 ± 1.42^i^	60.28 ± 3.60^gh^	8.23 ± 0.21^h^
C2	18.0 ± 0.12^efg^	68.95 ± 0.01^a^	0.19 ± 0.03	5.67 ± 0.01^a^	6.321 ± 0.500^j^	114.91 ± 0.83^a^	1.09 ± 0.31^j^	0.05 ± 0.01^k^
C3	19.0 ± 0.10^a^	61.95 ± 0.01^efg^	0.20 ± 0.06	4.50 ± 0.02^h^	9.037 ± 0.388^i^	88.84 ± 0.30^cd^	63.70 ± 4.00^fg^	6.12 ± 0.10^i^
C4	17.9 ± 0.06^fg^	68.72 ± 0.01^a^	0.17 ± 0.01	5.61 ± 0.02^b^	7.616 ± 0.906^ij^	91.65 ± 7.01^c^	21.16 ± 1.23^i^	0.05 ± 0.01^k^
C5	18.8 ± 0.10^b^	60.90 ± 0.02^g^	0.2 ± 0.04	4.51 ± 0.02^h^	7.958 ± 0.317^ij^	89.76 ± 5.16^cd^	67.79 ± 6.24^f^	11.95 ± 0.07^g^
C6	17.9 ± 0.10^efg^	63.29 ± 0.01^efg^	0.22 ± 0.02	4.66 ± 0.01^e^	7.496 ± 0.060^ij^	75.60 ± 6.08^ef^	63.55 ± 1.49^fg^	4.76 ± 0.07^j^
C7	18.0 ± 0.10^efg^	64.34 ± 0.01^def^	0.2 ± 0.03	4.71 ± 0.01^d^	7.056 ± 0.357^j^	74.24 ± 6.62^efg^	68.69 ± 1.24^ef^	4.43 ± 0.14^j^
C8	18.6 ± 0.06^c^	61.32 ± 0.01^fg^	0.2 ± 0.04	5.05 ± 0.01^c^	7.507 ± 0.222^ij^	54.40 ± 2.88^i^	57.81 ± 1.35^h^	6.09 ± 0.06^i^
C9	17.5 ± 0.06^h^	61.75 ± 0.01^efg^	0.22 ± 0.02	4.52 ± 0.06^h^	11.249 ± 0.693^h^	103.82 ± 4.12^b^	93.04 ± 3.60^a^	6.61 ± 0.14^i^
C10	16.2 ± 0.06^i^	65.22 ± 0.02^bc^	0.21 ± 0.02	4.46 ± 0.01^i^	8.928 ± 0.472^i^	84.31 ± 6.37^d^	83.36 ± 1.09^b^	4.37 ± 0.30^j^
C11	17.9 ± 0.10^efg^	62.52 ± 0.02^efg^	0.19 ± 0	4.63 ± 0.01^f^	14.366 ± 0.206^g^	102.49 ± 6.64^b^	95.32 ± 3.63^a^	4.12 ± 0.05^j^
C12	18.0 ± 0.10^efg^	61.61 ± 0.01^fg^	0.2 ± 0.01	4.60 ± 0.01^g^	14.713 ± 0.992^g^	90.33 ± 4.14^cd^	80.30 ± 1.46^bc^	8.70 ± 0.22^h^

*Note:* N stands for NAH, C stands for CAH. Values are presented as mean ± standard deviation (*n* = 3). Differences among groups were analyzed by one‐way ANOVA followed by Bonferroni correction. Values with different letters (a–q) in the same column are significantly different (*p* < 0.05).

Sugars are the dominant components of honey, accounting for 95%–99% of its dry weight. The total sugar content and composition of honey are primarily determined by the floral source and geographical origin of the nectar (Wu et al. 2025). Fructose and glucose are the primary sources of sweetness and serve as key indices for honey quality evaluation. Sucrose content is an indicator used to evaluate the maturity of honey. When the sucrose content is high, it is proved that the maturity of honey is low, and the sucrose in honey has not been transformed (Escuredo et al. 2014). The EU standard stipulates that (Council Directive 2001/110/EC of 20 December 2001 Relating to Honey 2001), the total content of glucose and fructose in honey is more than 60%, and the content of sucrose and maltose is < 5%. The total content of glucose and fructose in NAHs was 62.61%–66.26%, and the total content of glucose and fructose in CAHs was 60.90%–68.95%. Notably, an F/G ratio of 1.2–1.5 is a well‐documented characteristic of AH, as a higher relative fructose content inhibits sugar crystallization; this explains why the AH exhibit low crystallinity at room temperature (Escuredo et al. 2014). In addition, the sucrose content of NAH was 0.18%–0.23%, and the maltose content was 2.46%–3.31%. The content of sucrose in CAH was 0.17%–0.22%, and the content of maltose was 0.4%–3.09%. All sugar‐related indices of both NAH and CAH fully complied with the aforementioned EU standards (Council Directive 2001/110/EC of 20 December 2001 Relating to Honey 2001).

The pH value of honey could represent the stability and freshness of honey to a certain extent. The lower pH value indicates that the growth of microorganisms is inhibited (Majewska et al. 2019). The pH of NAH is between 4 and 5, and the pH of C1 and C2 honey is > 5, which is considered to be not mature enough.

Phenolic acids and flavonoids in honey are considered to be the main sources of antioxidant activity and are important substances for honey to play a biologically active role (Cucu et al. 2024). The TPC of NAH is 64.56–77.24 mg/100 g, and the TPC of CAH is 53.601–114.911 mg/100 g. The TFC of NAH is 17.873–22.627 mg/100 g. The TFC of CAH is 3.543–14.713 mg/100 g. Overall, the TPC and TFC values of NAH samples exhibited high uniformity, reflecting consistent bioactive component profiles across natural samples. By comparison, TPC and TFC varied significantly among different CAH brands. Specifically, two CAH brands (designated C1 and C8) had the lowest TPC and TFC values within the CAH group; given the established link between phenolic/flavonoid content and antioxidant capacity (Cucu et al. 2024), these two brands were inferred to possess weak antioxidant activity.

Amylase is an enzyme secreted by worker bees in the salivary gland and hypopharyngeal gland during honey maturation. It is considered to be a marker of honey freshness and its proper processing. It is also an important indicator of honey maturity, storage time and adulteration (Cucu et al. 2021). The amylase activity of NAH ranged from 14.318 to 28.720 mL/(g·h). The range of amylase activity of CAH are 0.046–11.948 mL/(g·h), and the CAH C2 and C4 were less than the EU standard 4 mL/(g·h), which may have an impact on honey‐processing. This defect may reflect an improper processing procedure (e.g., excessive thermal treatment, which denatures enzymes, or use of immature honey with incomplete enzyme accumulation).

Honey contains a variety of amino acids, of which proline is the dominant amino acid component. Proline content is widely recognized as a reliable basis for identifying honey maturity and detecting adulteration, as it accumulates gradually during honeybee‐mediated ripening and is typically depleted or diluted in adulterated products (Liu et al. 2025). At present, the export trade generally stipulates that the content of pure mature honey proline should not be less than 18 mg/100 g. The range of proline content of NAH is 73.02–80.311 mg/100 g. The range of proline content of CAH is 1.09–95.32 mg/100 g. Only C2 does not meet the standard.

In summary, the physicochemical indices of NAH consistently complied with both Chinese national honey standards and international benchmarks. All NAH samples exhibited uniformity across key quality parameters that confirmed their stable and high‐quality profile. In contrast, CAH showed heterogeneous quality, with two specific samples (C2 and C4) failing to meet standard requirements for critical indicators. Collectively, these physicochemical analyses demonstrate that NAH samples maintain consistently high‐quality, whereas CAH samples display significant quality variability, some samples meeting regulatory standards and others exhibiting deficiencies. This quality discrepancy between NAH and CAH likely stems from differences in raw material maturity or post‐harvest processing. The above experimental results provide a data‐driven basis for distinguishing high‐quality AH and guiding quality control in the honey industry.

### 3.2 UPLC‐Q‐Exactive Orbitrap‐MS Analysis of Components

**FIGURE 1:**
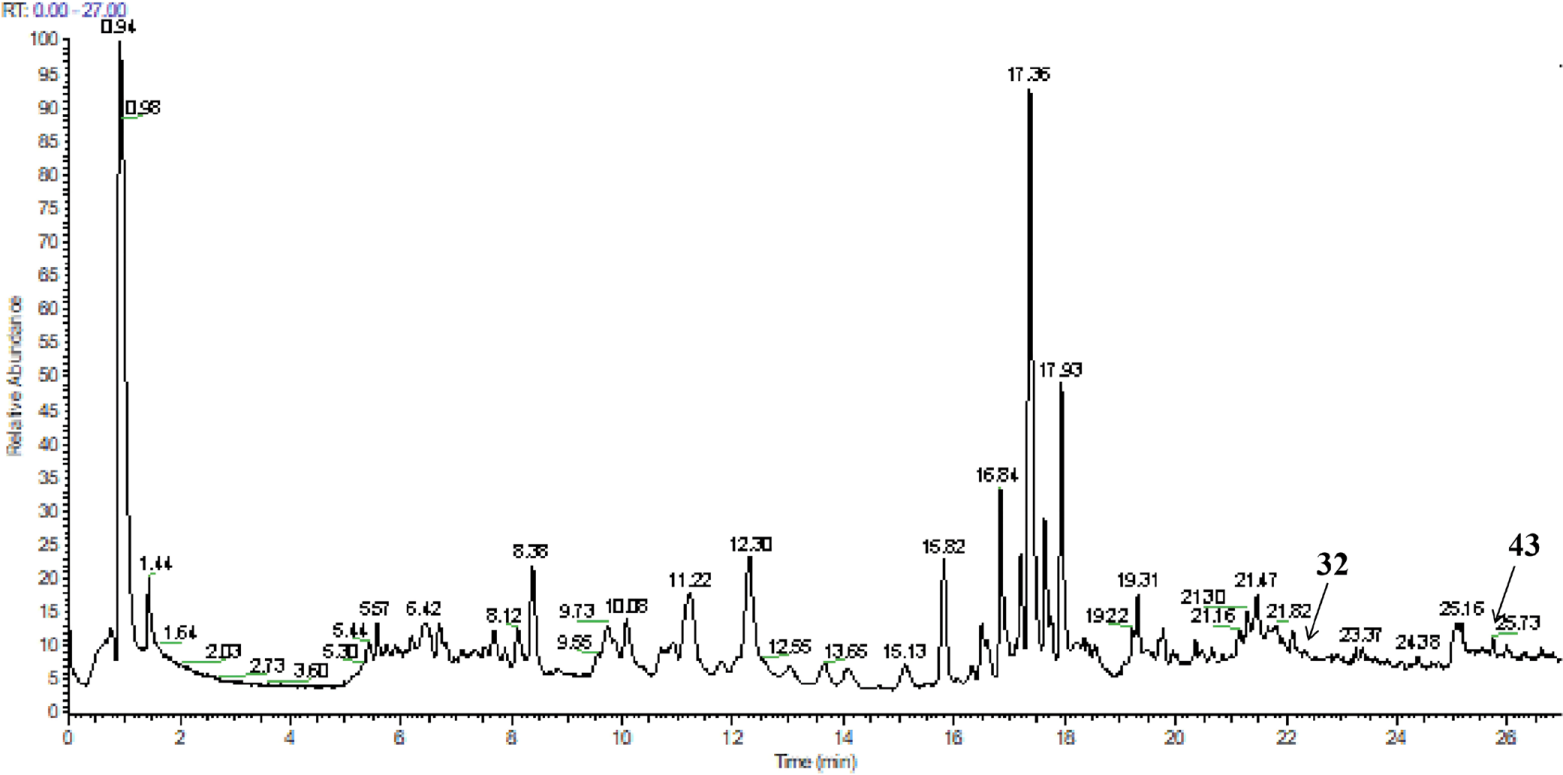
Total ion chromatogram (TIC) of AH samples obtained by UPLC‐QE‐MS in negative ion mode. 27: Hesperetin (*CAS: 520‐33‐2*), 36: Pinocembrin (*CAS: 480‐39‐7*).

Polyphenols are one of the most biologically active compound classes in honey, with flavonoids accounting for more than 50% of the total polyphenol content. As key secondary metabolites of plants, flavonoids and other polyphenols are directly associated with the functional properties of honey. For the qualitative and quantitative analysis of these compounds, the negative ion electrospray ionization (ESI^−^) mode of reverse‐phase LC–MS is widely recognized as one of the most robust and commonly used analytical methods, owing to its sensitivity and selectivity toward polar, acidic secondary metabolites. It is noteworthy that the composition of polyphenols and flavonoids in honey is closely related to its floral source and geographical origin, as these secondary metabolites are directly synthesized by plants and retain their species‐specific characteristics. This specificity enables polyphenols to serve as reliable tools for honey classification and authenticity verification, particularly for monofloral honey (Qi et al. 2025). Figure 1 shows the total ion chromatogram (TIC) of UPLC‐Q‐Exactive Orbitrap‐MS. A total of 50 compounds were identified from LC–MS (Table 2), which include 24 flavonoids, 20 phenolic acids, 4 fatty acids, 1 alkaloid, and 1 sesquiterpene. To screen for chemical markers capable of distinguishing between NAH and CAH, machine learning (ML) fitting and multivariate statistical methods (including PCA, OPLS‐DA, and VIP) were applied to the peak area data of 50 compounds.

**TABLE 2:** Identification of bioactive compounds in AH samples.

No.	RT	Compounds	Formula	[M‐H]^−^ (*m/z*)	PPM	MS/MS
**Phenolic acid**						
1	1.114	Trans‐4‐Hydroxycinnamic acid	C_9_H_8_O_3_	163.8497	0.2	163;145;119;93
2	1.133	4‐Hydroxybenzoic acid	C_7_H_6_O_3_	137.8968	−0.127	137;93;65;59
3	5.335	Protocatechuic acid	C_7_H_6_O_4_	153.0186	−0.209	153;109:108;91;81
4	5.636	Homovanillic acid	C_9_H_10_O_4_	181.0502		181;137;93;79;67
5	6.176	Salicylic acid	C_7_H_6_O_3_	137.0235	−0.127	137;93;65
6	6.31	2,4‐Dihydroxybenzoic acid	C_7_H_6_O_4_	153.0185	−0.209	153;109;91;81
7	6.486	Benzoic acid	C_7_H_6_O_2_	121.0285	−0.023	121;93;77;59
8	6.552	Vanillin	C_8_H_8_O_3_	151.039	0.216	151;136;123;108;95;92
9	6.57	2‐Hydroxyphenylacetic acid	C_8_H_8_O_3_	150.9	0.216	151;136;123;107
10	7.158	Gallic acid	C_7_H_6_O_5_	169.0505	−0.275	169;125;97;69
11	7.201	Caffeic acid	C_9_H_8_O_4_	179.0342	0.1	179;161;135;117;91;89;79
12	8.778	4‐Hydroxycinnamic acid	C_9_H_8_O_3_	163.0391	0.2	163;145;119;93;65
13	11.089	Isoferulic acid	C_10_H_10_O_4_	193.0499	−0.166	193;178;149;134;95;59
14	17.3532	Vanillyl alcohol	C_8_H_10_O_3_	153.0911	/	153;137;135;123;107
15	17.3535	Vanillic acid	C_8_H_8_O_4_	167.0704	0.108	167;152;123;108
16	23.6741	Ellagic acid	C_14_H_6_O_8_	301.0348	/	301;283;257;227;191;175
17	25.981	Caffeic acid phenethyl ester	C_17_H_16_O_4_	283.0963	0.063	283;179;161;135
**Flavonoid**						
18	7.407	Quercetin 3,4′‐diglucoside	C_27_H_30_O_17_	625.1431	/	463;445;301;283
19	8.804	Rutin	C_27_H_30_O_16_	609.1474	/	609;301;300;271
20	12.054	Nicotiflorin	C_27_H_30_O_15_	593.1509	0.011	593; 449, 285;284;255
21	15.965	Myricetin	C_15_H_10_O_8_	317.0666	0.03	317;289;151;125
22	17.832	Luteolin‐7‐O‐glucoside	C_21_H_20_O_11_	447.1942	0.035	447;284
23	17.982	Eriodictyol	C_15_H_12_O_6_	287.0567	−0.039	287;151;135;107
24	19.587	Hyperoside	C_21_H_20_O_12_	463.2558	0.002	463;301;271;255;179;151
25	21.6	Pinobanksin	C_15_H_12_O_5_	271.0616	0.012	271;253;243;151;107
26	22.148	5,7‐Dihydroxy‐2‐(3‐hydroxy‐4‐methoxyphenyl)chroman‐4‐one	C_16_H_14_O_6_	302.0753	0.128	301;286;151;136
27	22.152	Hesperetin	C_16_H_14_O_6_	301.0718	0.128	301;285;177;153;151;136;108
28	22.363	Genistein	C_15_H_10_O_5_	269.1092	−0.173	269;225;133;117
29	23.6742	Morin	C_15_H_10_O_7_	301.2005	0.08	301;271;227;151;125;107
30	23.842	Formononetin	C_16_H_12_O_4_	267.0571	0.067	267;252;223;195;135;132
31	24.026	Afzelin	C_21_H_20_O_10_	431.2265	0.07	431;284;285;151
32	24.193	Quercetin	C_15_H_10_O_7_	300.1733	0.08	301;273;257;227;178;151;107
33	24.21	Chrysoeriol	C_16_H_12_O_6_	299.1329	−0.038	299;284;256;227;151;133;107
34	25.31	Naringenin	C_15_H_12_O_5_	270.1927	0.012	271;151;119;107
35	25.527	Luteolin	C_15_H_10_O_6_	285.0794	0.136	285;267;255;151;133;107
36	25.568	Pinocembrin	C_15_H_12_O_4_	255.0673	0.07	255;227;151;107
37	26.233	Isorhamnetin	C_16_H_12_O_7_	315.2179	−0.082	315;300;283;271;227;151;107
38	26.443	Chrysin	C_15_H_10_O_4_	253.0657	−0.127	253;209;145;143;119;107
39	27.86	Glycitein	C_16_H_12_O_5_	283.192	0.012	283;268;240;239;211
40	27.889	Kaempferol	C_15_H_10_O_6_	285.208	0.136	285;257;255;241;239;227;151;117
41	30.363	Diosmetin	C_16_H_12_O_6_	299.2593	−0.038	299;284;256;227;151;133;107
**Fatty acids**						
42	7.973	3‐Dehydroshikimic acid	C_7_H_8_O_5_	171.0631	0.02	171;153;127;109;97
43	16.74	10‐HDA	C_10_H_18_O_3_	185.1178	−0.096	185;139;137;123;109;59
44	17.3536	Phenoxyacetic acid	C_8_H_8_O_3_	151.0751	0.216	151;136;133;107;93
45	22.304	Corchorifatty acid F	C_18_H_32_O_5_	327.2177	0.008	327;229;221;211;171
46	26.691	(±)9‐HpODE	C_18_H_32_O_4_	311.2202	0.055	311;293;193;183;171
**Quinoline alkaloids**						
47	5.793	Kynurenic acid	C_10_H_7_NO_3_	188.0351	−0.087	188;144;102
**Phenylpropanoid**						
48	6.665	Chlorogenic acid	C_16_H_18_O_9_	353.0764	−0.015	353;191;173;85
**Coumarins**						
49	17.3533	4‐Hydroxycoumarin	C_9_H_6_O_3_	161.0947	−0.108	143;133;117;115;99
**Sesquiterpene**						
50	17.354	Abscisic acid	C_15_H_20_O_4_	263.1291	0.067	263;219;203;153;137

### 3.3 Machine Learning

**FIGURE 2:**
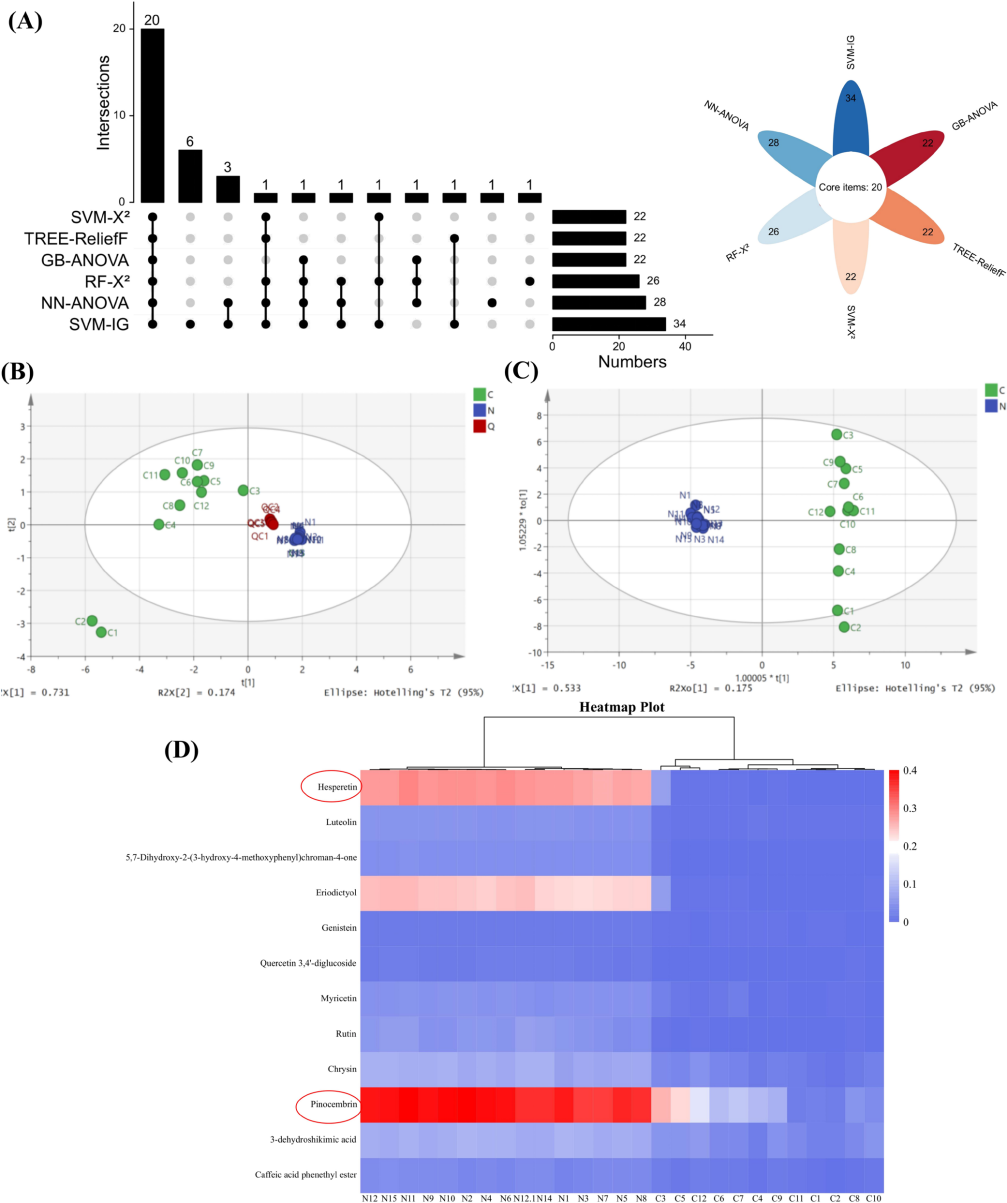
(A) ML analysis to select key features (venn diagram indicates intersection of different models, bar chart indicates the number of common elements across sets, dot plot indicates the presence/absence of sets in intersections). (B) PCA score plot. (C) OPLS‐DA score plot. (D) Heat map of the relative content of components with VIP > 1 in honey. C, CAH; N, NAH; Q, Quality control.

Machine learning (ML) can learn intricate patterns from data and continuously refine its algorithms through experience, thereby accurately classifying and predicting data while mitigating the subjectivity inherent in traditional data analysis methods (Jordan and Mitchell 2015; Gonzalez Viejo et al. 2019). ML has been widely applied in Traditional Chinese Medicine (Nie et al. 2025). To accurately distinguish NAH and CAH, the selection of key characteristic components was performed by combining feature selection algorithms with machine learning methods based on UPLC‐QE‐MS detection data (15 NAH and 12 CAH samples). And five classical machine learning algorithms, including SVM (Support Vector Machine), NN (Neural Network), RF (Random Forest), TREE (Decision Tree) and GB (Gradient Boosting), were combined with four feature selection methods IG, ANOVA, χ^2^ and ReliefF to construct combined models. As shown in Table 3, the AUC values of all combined models were ≥ 0.986 and the CA values were ≥ 0.965. Among them, the SVM‐IG and GB‐ANOVA models achieved a perfect score of 1.0 for all evaluation indices, which were higher than the conventional qualification criteria for model assessment in foodomics (AUC ≥ 0.8, CA ≥ 0.75). These results indicate that the constructed models possess excellent classification performance and predictive power. Figure 2A shows the key characteristic components identified by integrating multiple feature selection algorithms and machine learning models. A Venn diagram is used to present the intersection of important features derived from four feature ranking methods: IG, ReliefF, X^2^, and ANOVA. The bar chart displays the frequency at which each component was selected by different models, while the scatter plot reflects the presence or absence of each component in various feature subsets. A total of 20 stable and important characteristic components were identified in this study, including: Luteolin, Hesperetin, 5,7‐Dihydroxy‐2‐(3‐hydroxy‐4‐methoxyphenyl)chroman‐4‐one, Eriodictyol, Genistein, Quercetin 3,4′‐diglucoside, Myricetin, Rutin, Chrysin, Caffeic acid phenethyl ester, Naringenin, 3‐dehydroshikimic acid, Pinocembrin, Luteolin‐7‐O‐glucoside, Vanillin, Isoferulic acid, Pinobanksin, Chlorogenic acid, 10‐HDA, Diosmetin. Validation results confirmed that these selected characteristic compounds can effectively distinguish quality differences among NAH and CAH, and are applicable for quality control purposes (Table 3).

**TABLE 3:** Model evaluation results based on feature subsets.

	AUC	CA	F1	Prec	Recall	MCC
SVM‐IG	1.000	1.000	1.000	1.000	1.000	1.000
NN‐ANOVA	0.986	0.965	0.934	0.978	0.971	0.942
RF‐X^2^	0.998	0.998	0.927	0.969	0.971	0.942
SVM‐X^2^	1.000	1.000	0.944	0.975	0.938	0.898
TREE‐ReliefF	0.999	0.983	0.986	0.929	0.991	0.889
GB‐ANOVA	1.000	1.000	1.000	1.000	1.000	1.000

Abbreviations: AUC, area under the curve; CA, classification accuracy; F1, F1 score; GB, gradient boosting; MCC, Matthews correlation coefficient; NN, neural network; Prec, precision; Recall, recall rate; RF, random forest; SVM, support vector machine; TREE, decision tree.

### 3.4 Principal Component Analysis

The mass spectrometry data of 15 NAH, 12 CAH and 5 quality control (QC) samples were imported into R 3.2.5 software for calibration processing, and the corrected data were imported into SIMCA14.1 for principal component analysis (PCA) to further study the situation of AH from different sources. It can be seen from the PCA plot (Figure 2B), the quality control (QC) samples clustered closely around the plot center. This observation confirms not only the reproducibility and stability of the LC–MS analytical platform but also the reliability of the dataset, it indicates that the chemometric results were not affected by instrumental sequence drift. The total three principles components can explain 93.5% of the total variance, of which the first principal component (PC1) represented 73.1%, and the next two principal components represented 17.4% and 0.29%, respectively. As shown in the PCA score plot (Figure 2B), The two sources of honey could be clearly distinguished. CAH were distributed in the negative axis of PC1 and NAH were distributed in the positive axis of PC1.

Hierarchical cluster analysis (HCA) was performed to describe the overall proximity between honey samples. The Euclidean distance was used as a distance measure to calculate the sample similarities between the honey samples, and the parameters of the clustering algorithm and linkage rule were set as Ward's and hierarchical values, respectively, set the clustering threshold to an Euclidean distance of 15. Figure S1 shows the results of HCA as a dendrogram. The algorithm has successfully grouped all the honey samples into two clusters, and the HCA results were consistent with the PCA results.

### 3.5 Orthogonal Partial Least Squares Discriminant Analysis

The orthogonal partial least squares discriminant analysis (OPLS‐DA) model was constructed by paired analysis of 15 NAH and 12 CAH samples. The results had high goodness of fit (R2Y = 0.995) and predictive ability (Q2 = 0.979) (Figure 2C), indicating that the identified components had correct separation and acceptable predictability for honey from different sources. Cross validation was evaluated using a permutation test with 200 cycles, where R2 and Q2 were calculated as the goodness of fit and the predictive capability of the model, respectively. The R2 and Q2 generated by the left random arrangement were 0.391 (> 0) and −0.66 (< −0.05), respectively, which were smaller than the original values of the right side, indicating that the model was predictive and reliable, and there was no over‐fitting (Figure S2).

The significance of variables was evaluated using the variable importance in the projection (VIP) method (Figure S3 and Table S1). The components obtained based on the condition that the VIP > 1.2 and the *p* < 0.05 were intersected with the components identified through machine learning, resulting in a total of 12 components. Combined with the heat map analysis (Figure 2D), it was found that among the 12 components, the contents of two components, hesperetin (*p* = 8.11745E−27) and pinocembrin (*p* = 1.57E−12), showed significant differences between the NAH and CAH groups, so they were selected as discriminative markers.

Ultimately, pinocembrin and hesperetin were selected as ideal chemical markers for identifying the natural origin of acacia honey—these compounds can help distinguish between NAH and CAH.

### 3.6 Assessment of Vitro Immune Activity the Proliferation of Spleen Lymphocytes Analysis

Lymphocyte is the body's immune active cells and an important part of specific immunity. Spleen is the largest peripheral immune organ. It is an important place for T and B lymphocytes to settle and produce an immune response after antigen stimulation (Wang et al. 2023; Zhang et al. 2025). It is closely related to cellular immunity and humoral immunity. Concanavalin A (Con A) and Lipopolysaccharide (LPS) are mitogens of T and B lymphocytes respectively, which could promote the proliferation of lymphocytes (Li 2023). Therefore, the effect of the drug on the immune level of the body could be judged according to the effect of the drug on the proliferation ability of lymphocytes.

**FIGURE 3:**
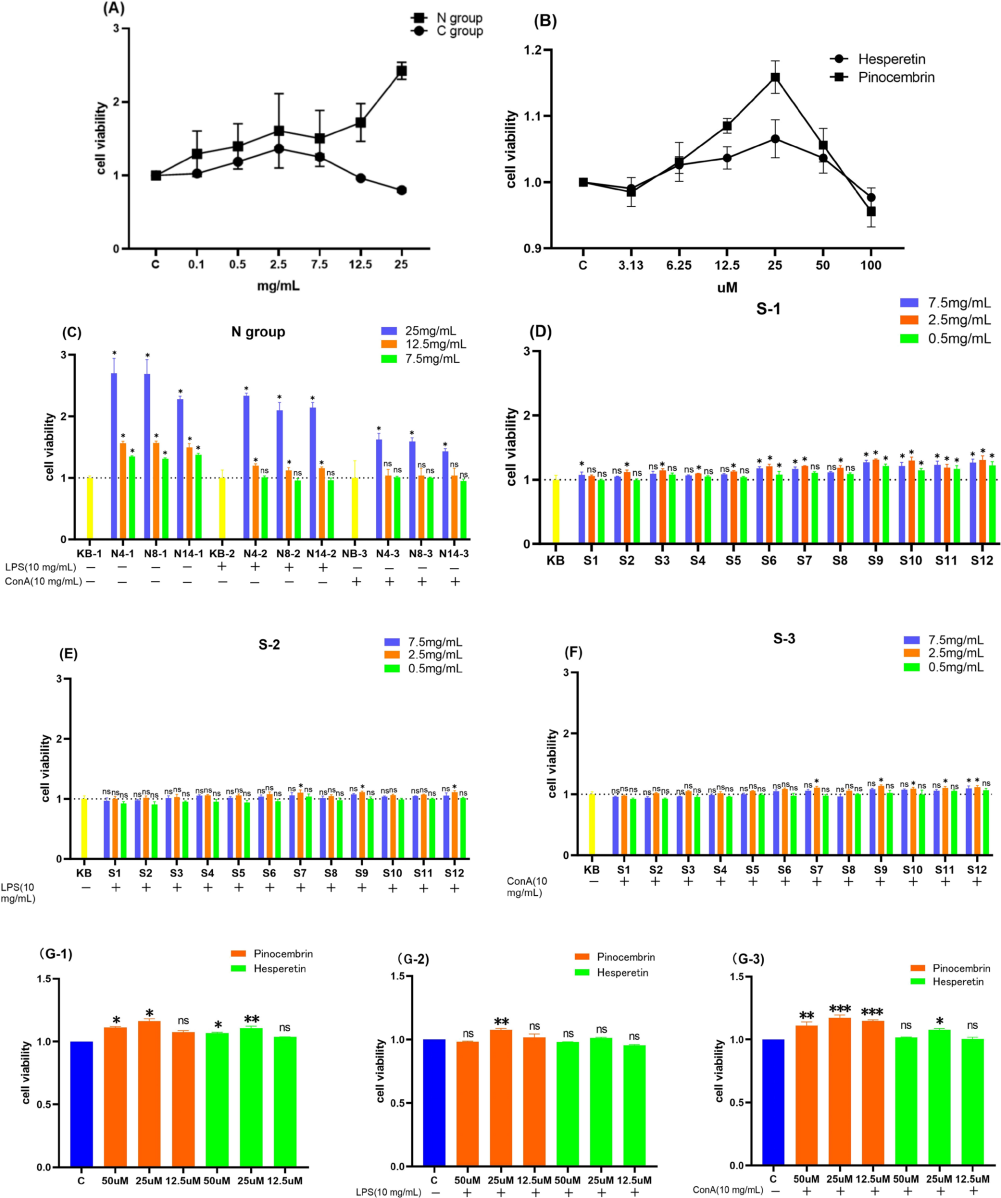
Effect of honey on the proliferation of mouse spleen cells. (A) The effect of 0.1–25 mg/mL concentration of NAH and CAH on the proliferation of spleen cells. (B) The effect of 0.2–100 μM concentration of the marker on the proliferation of spleen cells. (C) The effects of NAH combined with LPS and ConA on the proliferation of mouse splenocytes. (D–F) The effects of CAH combined with LPS and ConA on the proliferation of mouse splenocytes. (G) Effects of chemical markers and their combination with LPS and ConA on the proliferation of mouse splenocytes. N: NAH; C: CAH. Values are expressed as mean ± SD (*n* = 3). Statistical comparisons were performed using one‐way ANOVA with Bonferroni correction. *p < 0.05, **p < 0.01, ***p < 0.001.

The above experimental results showed that the quality of NAH was uniform, so we randomly selected 3 NAH and 12 CAH for experiments (The experimental results are shown in Figure 3). Firstly, the optimal concentrations of CAH and NAH were determined by CCK‐8 experiment to be 7.5, 2.5, 0.5 mg/mL and 25, 12.5, 7.5 mg/mL, respectively; pinocembrin and hesperetin were 50 μM, 25 μM, and 12.5 μM. In the experimental range, compared with the control group, the NAH and the CAH group could effectively promote the proliferation of spleen lymphocytes in mice, and had a synergistic effect with Con A and LPS on the proliferation of T and B lymphocytes. The proliferation rate of CAH ranged from 105.333% to 130.767%, and the proliferation rate of NAH ranged from 227.767% to 269.967%. The proliferation effect of CAH 2.5 mg/mL and NAH 25 mg/mL was the most significant. These results indicate that NAH exhibits stronger immune‐enhancing activity than CAH. Both pinocembrin and hesperetin significantly promoted the proliferation of splenic lymphocytes (*p* < 0.05), and their synergistic effects with Con A were stronger than those with LPS. Furthermore, within the tested concentration range, pinocembrin exhibited stronger immunomodulatory activity than hesperetin.

### 3.7 Protective Effect of Chemical Markers on LPS‐Induced RAW264.7 Cell Inflammation Model

Macrophages and neutrophils are the key mediators of the inflammatory response, and play a crucial role in initiating, maintaining, and eliminating inflammation by phagocytizing or producing cytokines (such as pro‐inflammatory tumor necrosis factor‐α). Macrophages secrete NO after activation, which plays an important role in activating various immune responses (Ahmad et al. 2020).

**FIGURE 4:**
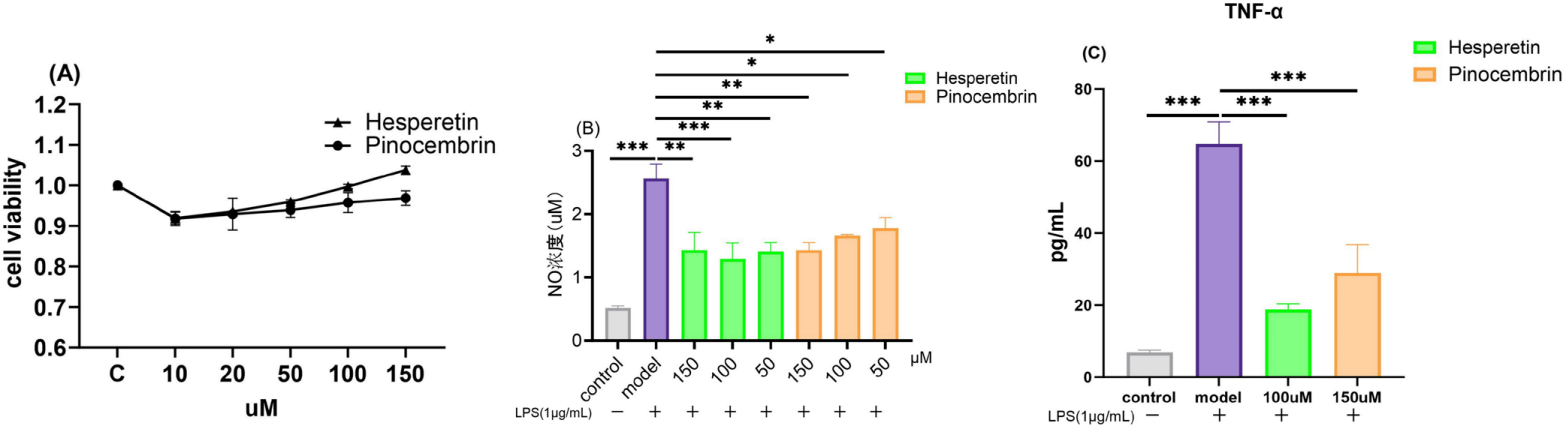
Effects of honey chemical markers on RAW264.7 cells (A) Cytotoxicity of 10–150 μM markers on RAW264.7 cells. (B) Nitrite levels were determined using the Griess method. (C) The secretion of TNF‐α was measured by ELISA method. The values are expressed as mean ± SD (*n* = 3). Values are expressed as mean ± SD (*n* = 3). Statistical comparisons were performed using one‐way ANOVA with Bonferroni correction. *p < 0.05, **p < 0.01, ***p < 0.001.

The cytotoxicity of the quality markers to macrophage RAW264.7 was evaluated by CCK‐8 assay. As presented in Figure 4A, in the experimental range, there was no significant cytotoxicity to the cells. The maximum concentration with only significantly promoting proliferation was selected as the concentration of administration (50–150 μM). Figure 4B shows the effect of 50–150 μM concentration on the secretion of NO in RAW264.7 cells. Compared with the model group, both markers could significantly inhibit the secretion of NO, and pinocembrin showed a significant dose‐effect relationship. Figure 4C showed that hesperetin and pinocembrin could significantly inhibit the secretion of cytokine TNF‐α; the dose and effect are consistent with those described in the article (Navia et al. 2026). Above results suggested that the marker had a significant protective effect on the LPS‐induced inflammatory model. Moreover, the inhibitory effects on NO and TNF‐α secretion exhibited a clear dose‐dependent manner.

### 3.8 Correlation Analysis

**FIGURE 5:**
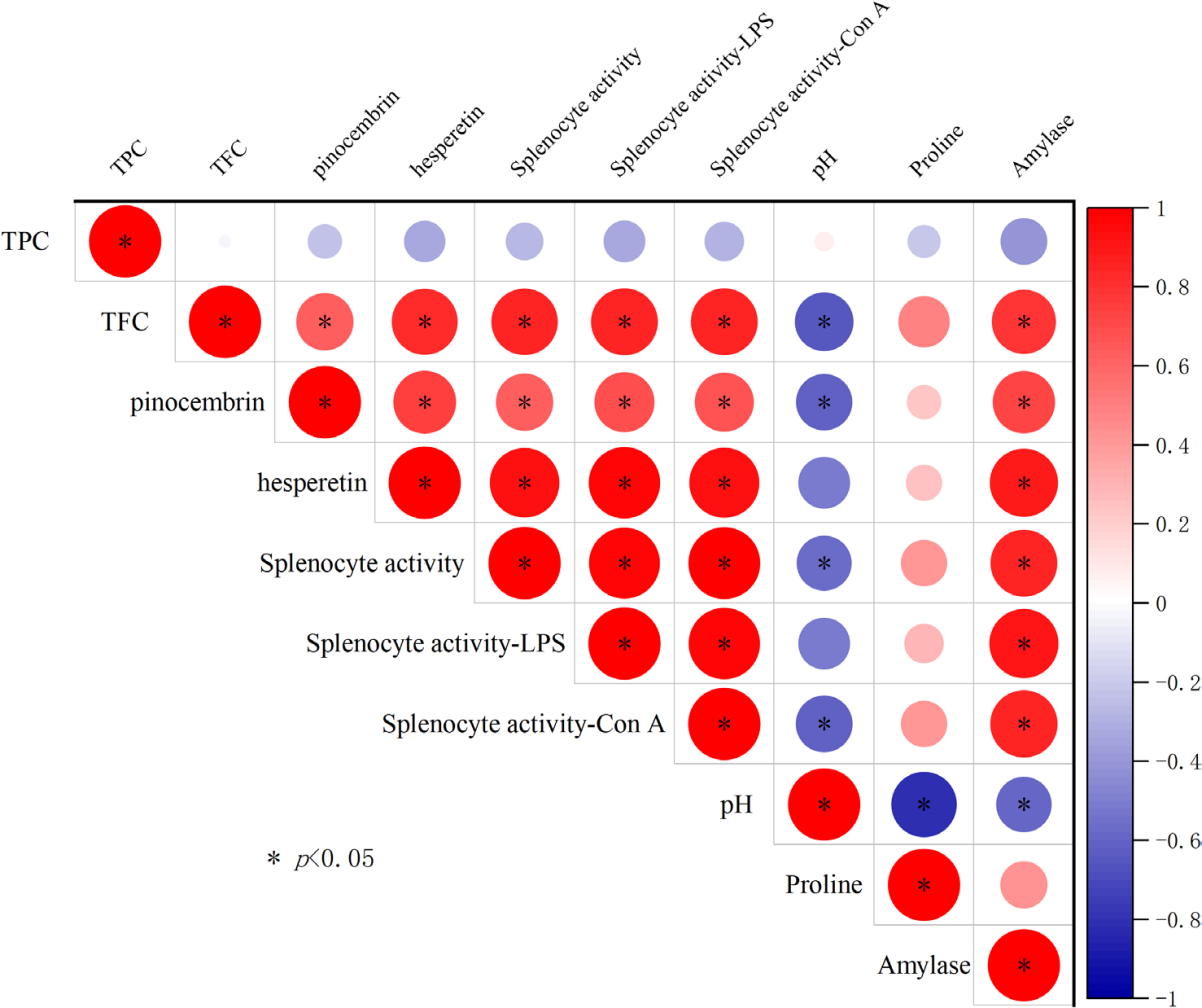
Correlation analysis of honey. Statistical significance was determined by Pearson correlation analysis with Bonferroni correction. *p < 0.05.

The physicochemical characteristics and characteristic markers of AH on the proliferation activity of mouse spleen lymphocytes were studied. The strength of the linear relationship between each pair of variables was obtained using the Pearson correlation test, using *p < 0.05 as the standard for statistically significant correlation. According to the correlation heat map in Figure 5, proliferation‐promoting activity showed a good positive correlation with TFC and two chemical markers; their correlation coefficients were 0.843, 0.848, and 0.855 (TFC), respectively; 0.628, 0.683, 0.668 (pinocembrin); 0.926, 0.969, 0.933 (hesperetin) (*p < 0.05); and show a good negative correlation with pH value. The correlation coefficients were −0.575, −0.507, −0.614. Moreover, TFC could directly affect the pH value of honey, indicating that TFC and markers are the key functional components affecting the immunomodulatory effect of AH.

### 3.9 Network Pharmacology Analysis

Two characteristic markers, hesperetin and pinocembrin, are core components in AH that possess both discriminative and bioactive properties. Therefore, network pharmacology analysis was performed on these two components to screen the core targets and signaling pathways through which the two markers exert immunomodulatory effects (anti‐inflammatory and immune‐enhancing activities), reveal the “multi‐component, multi‐target, multi‐pathway” mechanism of action of the markers, establish the association between the characteristic markers of AH and immune activity, and verify the rationality of their role as “dual markers for quality and function” to clarify the purpose of network pharmacology analysis and its association with AH.

**FIGURE 6:**
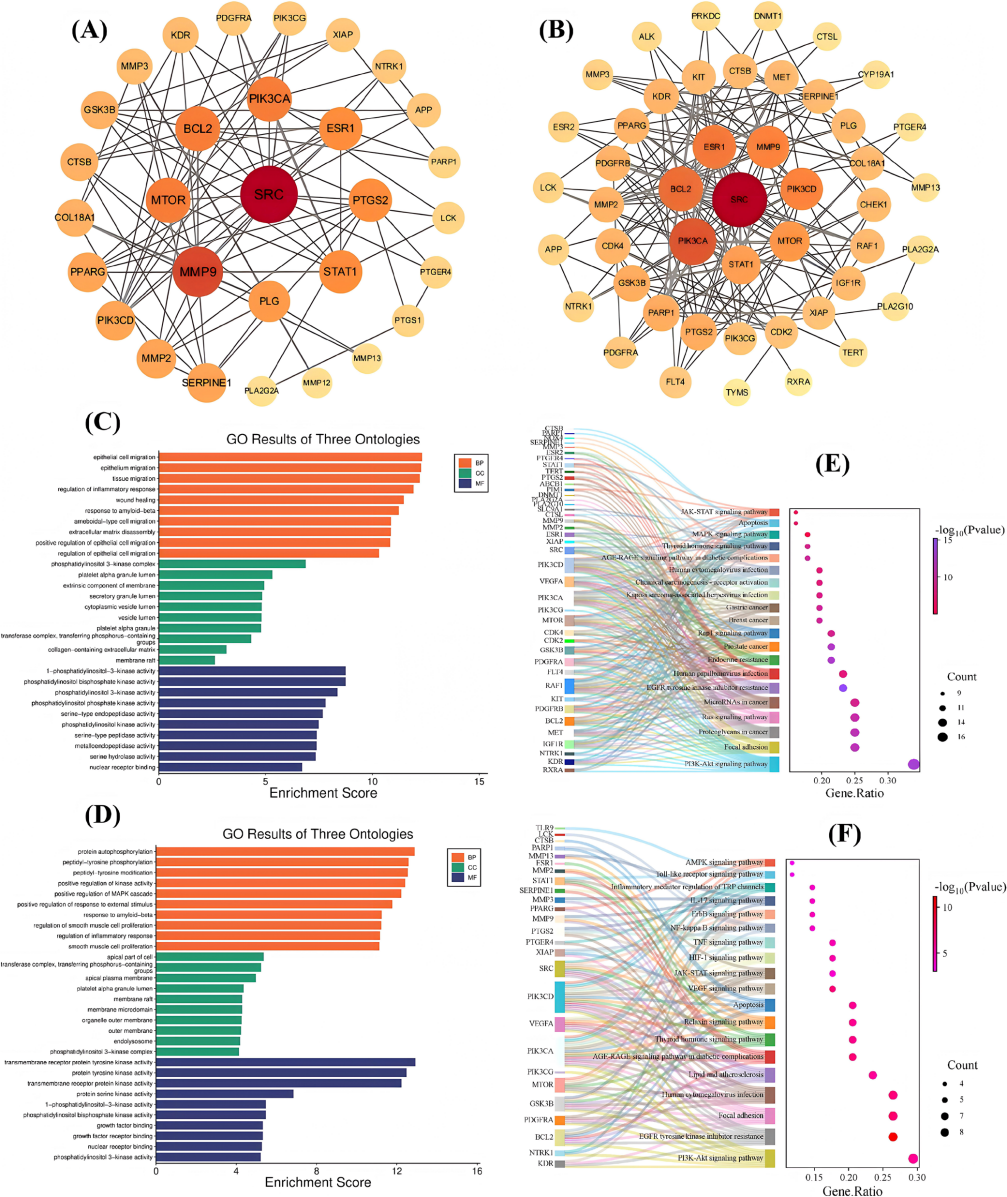
Network analysis of chemical markers (A, B). The PPI network of markers, inflammation and enhance immunity targets constructed via STRING. (C, D) The GO enrichment analysis of inflammation and enhance immunity targets. (E, F) The KEGG pathway enrichment analysis of inflammation and enhance immunity targets.

Using the Swiss Target Prediction database with *Homo sapiens* as the species restriction, 147 potential common targets of hesperetin and pinocembrin were predicted. According to relevance score > 5, 735 inflammatory targets and 1176 enhanced immunity targets were identified from the Genecards and OMIM databases. The intersection analysis showed 36 and 57 common targets, respectively (Figures S4, S5), which represent the potential molecular targets for anti‐inflammatory and enhanced immunity markers. The key marker‐related targets and disease‐related targets were imported into Cytoscape 3.9.0 for visualization, thereby constructing the ppi network diagram of common targets (Figure 6A,B) and the “component ‐target‐disease” network (Figures S6, S7). Notably, a single active ingredient may correspond to multiple targets, and different active ingredients may also share the same target, indicating that the drug plays a role in the disease through a multi‐component, multi‐target mechanism. In order to clarify the key targets and signaling pathways of markers in anti‐inflammation and enhanced immunity, gene ontology (GO) analysis (Figure 6C,D) and KEGG pathway analysis (Figure 6E,F) were performed on the common targets using the DAVID database.

SRC, MMP9, PTGS2, BCL2, ESR1, and MMP2 were identified as the core targets of anti‐inflammation (Figure 6A). GO enrichment analysis showed that biological process (BP) mainly focused on the regulation of inflammatory response, response to oxidative stress, and positive regulation of cytokine production. Cell components (CC) were concentrated in platelets‐related structures, secretory granule lumen, and vesicle structures. Molecular function (MF) mainly focused on hydrolase activity, coagulation, and complement system‐related activity, as well as molecular functions that support cell survival, growth, metabolism, and blood glucose homeostasis. The top ten BP, CC, and MF items are shown in Figure 6C. KEGG analysis showed the top 20 pathways with the highest enrichment values (Figure 6E). Notably, the anti‐inflammatory mechanisms of these markers primarily involve the regulation of seven classic signaling pathways related to inflammation and cell survival: the PI3K‐Akt signaling pathway, apoptosis pathway, adhesion molecule signaling pathway, JAK–STAT signaling pathway, TNF signaling pathway, NF‐κB signaling pathway, and MAPK signaling pathway.

SRC, BCL2, ESR1, MMP9, PIK3CA, MTOR, PPARG, PTGS2, and CDK4 were identified as the core targets of enhanced immunity (Figure 6B). GO enrichment analysis showed that biological process (BP) mainly focused on protein autophosphorylation, peptidyl−tyrosine modification, positive regulation of MAPK cascade, and regulation of inflammatory response. Cell components (CC) were concentrated in the apical part of the cell, transferase complex, transferring phosphorus‐containing groups, and apical plasma membrane. Molecular function (MF) is mainly focused on transmembrane receptor protein tyrosine kinase activity, protein tyrosine kinase activity, transmembrane receptor protein kinase activity, protein serine kinase activity, etc. It regulates cell proliferation, differentiation, apoptosis, signal transduction, immune regulation, metabolism, movement, membrane transport, tumorigenesis, etc. The top ten BP, CC, and MF items are shown in Figure 6D. KEGG analysis showed the top 20 pathways with the highest enrichment values (Figure 6F). The results indicated that the mechanism of markers in the treatment of enhanced immunity mainly involved the PI3K‐Akt signaling pathway, Focal adhesion pathway, Proteoglycans in cancer pathway, Ras signaling pathway, MicroRNAs in cancer pathway, and EGFR signaling pathway regulation.

Notably, the PI3K‐Akt pathway was consistently identified as the most enriched pathway in both analyses and exhibited functional overlap that links anti‐inflammatory and immune‐enhancing effects. Based on this dual regulatory role of simultaneously balancing inflammatory responses and enhancing immune cell function, the PI3K‐Akt signaling pathway is regarded as one of the key common pathways through which pinocembrin and hesperetin mediate their biological activities, particularly in acacia honey.

### 3.10 Molecular Docking

**FIGURE 7:**
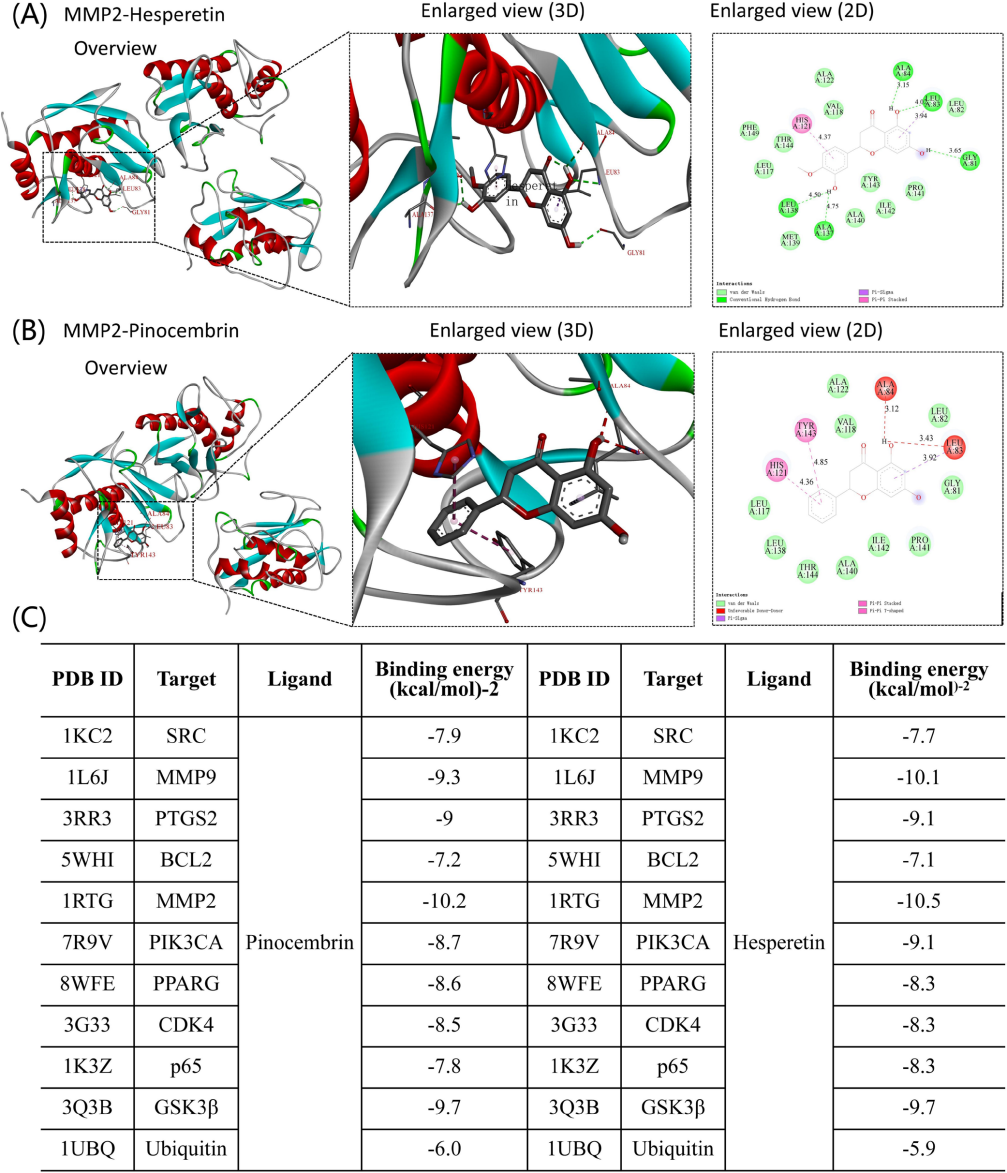
(A, B) Molecular docking of MMP2 complexed with two chemical markers; (C) Binding energy of chemical markers with key targets.

Through network pharmacology, GO and KEGG analysis, the main pathways and targets for the markers to exert anti‐inflammatory and enhance immunity were determined. To validate the network pharmacology predictions and further elucidate the molecular mechanisms underlying chemical markers' anti‐inflammation and enhance immunity effect, molecular docking was performed using CB‐Dock2. The binding energies between ligands (hesperetin/pinocembrin) and key targets were calculated by the semi‐empirical free energy calculation method. Lower binding energies generally indicate stronger ligand–receptor interactions and more stable conformations in molecular docking analysis. Binding energies below −5.0 kcal/mol are commonly considered indicative of stable binding, whereas values below −7.0 kcal/mol suggest strong binding affinity. Binding energies lower than −9.0 kcal/mol are often interpreted as reflecting very strong interactions (Pantsar and Poso 2018). For the identification and validation of interaction types, Discovery Studio software was used to analyze the optimal conformation of molecular docking, and interaction types such as van der Waals forces, hydrogen bonds, and π‐π stacking were identified (Deng et al. 2024). The docking of markers with key targets for anti‐inflammatory and immune enhancement can be seen in Figure 7C. Except for SRC and p65, which are derived from *Mus musculus* , all other target proteins are derived from *Homo sapiens* . Molecular docking analysis showed that there was a strong binding interaction between the marker and all key targets, and the binding energy was lower than −7.0 kcal/mol, indicating that the docking conformation was strong. Among them, the binding energies with MMP2 are −10.5 and −10.2 kcal/mol respectively, showing the strongest binding force, which proves that MMP2 is the key target for the two markers to exert immunomodulatory/anti‐inflammatory effects, and the molecular docking pattern diagram of MMP2 and markers were shown in Figure 7A,B. A detailed analysis of the binding modes identified various types of interactions, van der Waals, Pi‐Sigma, Conventional Hydrogen Bond, Pi‐Pi stacked, Unfavorable Donor‐Donor and hydrogen bonds.

To evaluate binding specificity, an unrelated globular protein (ubiquitin, PDB ID: 1UBQ) was used as a non‐target control. Ubiquitin is a highly conserved, structurally stable protein widely used as a model system in structural and molecular interaction studies. Its compact fold and minimal surface cavities, along with the absence of well‐defined small‐molecule binding pockets, make it suitable as a background control for assessing nonspecific interactions (Ganesan and Kiyokawa 2025; Loh et al. 2024). The results showed that the binding energies of hesperetin and pinocembrin with ubiquitin were −5.9 kcal/mol and −6.0 kcal/mol, respectively, both of which were higher than the binding energy between the marker and the core target. This proves that the binding of the two markers to the core target is a specific binding.

### 3.11 Molecular Dynamics Simulation

**FIGURE 8:**
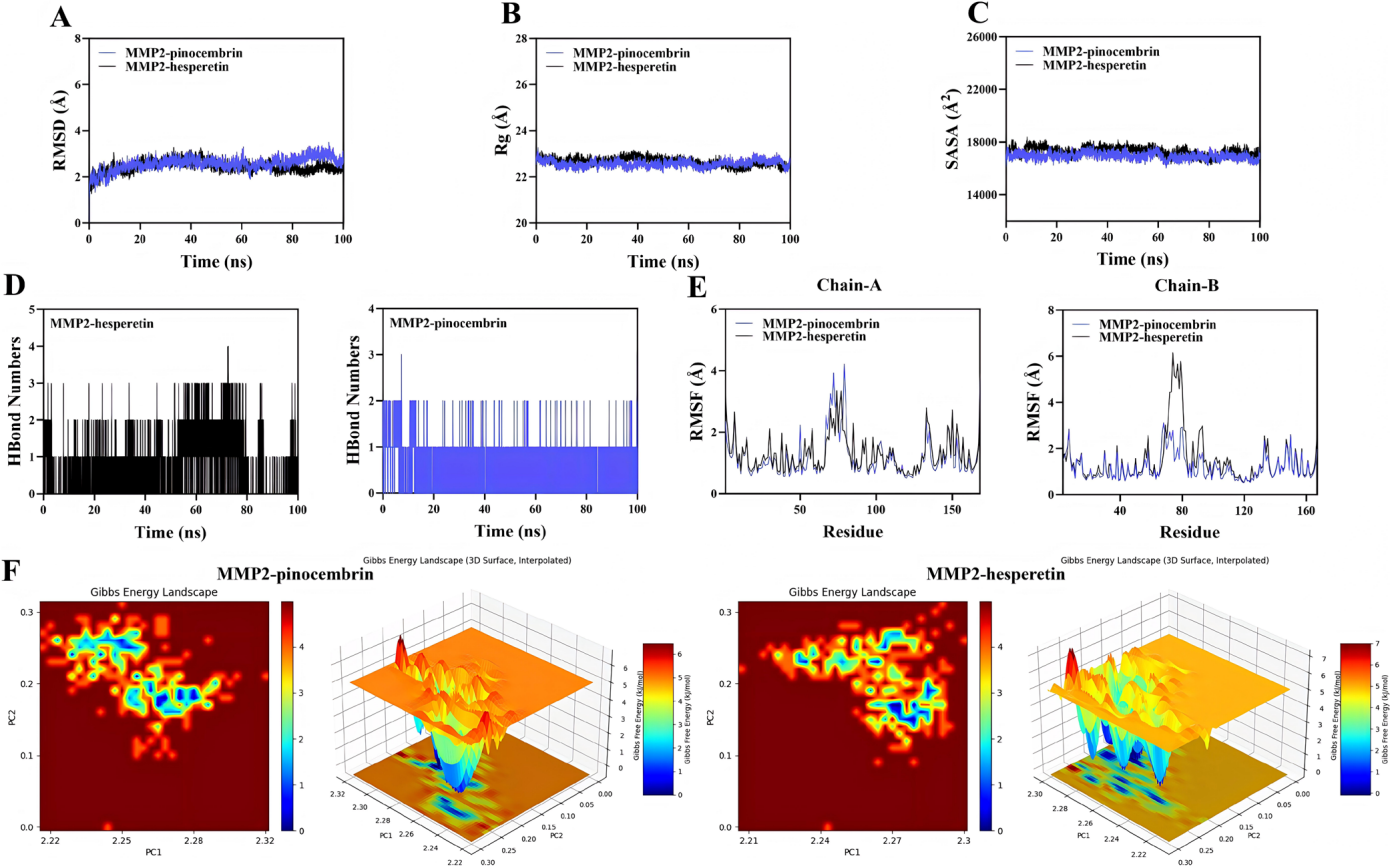
Molecular dynamics simulation of protein‐ligand complexes. (A) RMSD values of protein‐ligand complexes over time (B) Rg values (C) SASA values (D) HBonds values (E) RMSF values (F) Free Energy Landscape.

The root‐mean‐square deviation (RMSD) is a good indicator for evaluating the conformational stability of proteins and ligands, as well as the degree of deviation of atomic positions from their initial positions. A smaller deviation indicates better conformational stability (Wei et al. 2025; Zhang et al. 2022). Therefore, RMSD was used to assess the equilibrium of the simulation system. As shown in Figure 8A, the MMP2‐pinocembrin complex fluctuated stably between 10 and 80 ns, showing a slight upward trend in the late stage of movement, but always fluctuated below 3 Å. The MMP2‐hesperetin complex system reached equilibrium after 10 ns and finally fluctuated around 2.4 Å. Therefore, the small molecules pinocembrin and hesperetin both exhibited high stability when bound to the MMP2 target protein, respectively.

The radius of gyration (Rg) is used to describe changes in the overall structure and characterize the compactness of the protein structure (Wei et al. 2025; Zhang et al. 2022). The MMP2‐pinocembrin and MMP2‐hesperetin complexes remained stable in fluctuation during the simulation, indicating that no obvious expansion or contraction occurred in the small molecule‐target protein complexes throughout the trajectory (Figure 8B).

The Solvent‐Accessible Surface Area (SASA) is an indicator used to evaluate the surface area of proteins (Wei et al. 2025). In this simulation, the solvent‐accessible surface area between the target protein and small molecules was calculated (Figure 8C). The results showed that after the binding of MMP2‐pinocembrin and MMP2‐hesperetin (receptor‐ligand binding), there was no significant change in the SASA of the complexes, which further indicates that the small molecules can achieve stable binding with the target protein.

Hydrogen bonds play an important role in the binding between ligands and proteins (Wei et al. 2025). The number of hydrogen bonds between small molecules and the target protein during the dynamic process is shown in Figure 8D. For the MMP2‐pinocembrin complex, the number of hydrogen bonds ranges from 0 to 3, and in most cases, there is approximately 1 hydrogen bond in the complex. For the MMP2‐hesperetin complex, the number of hydrogen bonds ranges from 0 to 4, and in most cases, there are about 2 hydrogen bonds in the complex. These results indicate that there is a good hydrogen bond interaction between the ligands and the target protein.

The Root Mean Square Fluctuation (RMSF) can indicate the flexibility of amino acid residues in proteins (Zhang et al. 2022). As shown in Figure 8E, the RMSF values of the MMP2‐pinocembrin complex are relatively low (mostly below 4 Å), and the RMSF values of the MMP2‐hesperetin complex are also relatively low (mostly below 6 Å). Therefore, both complexes exhibit low flexibility and high stability.

The Free Energy Landscape (FEL) is shown in Figure 8F, presenting the free energy surface results of the complexes. Color mapping is used in the figure to represent different free energy values: the red regions indicate higher free energy, while the blue regions represent lower free energy. The X‐axis represents PC2 (Root Mean Square Deviation) with the unit of nanometers, and the Y‐axis represents PC1 (Radius of Gyration), also in nanometers. It can be observed from the figure that the MMP2‐pinocembrin complex has a lower free energy when PC2 is 0.17 and PC1 is 2.295, and the MMP2‐hesperetin complex has a lower free energy when PC2 is 0.15 and PC1 is 2.285. These conformations are likely to be more stable.

In summary, both the MMP2‐pinocembrin and MMP2‐hesperetin complex systems exhibit stable binding, and the complexes have good hydrogen bond interactions. Therefore, the small molecules bind well with the target protein.

## 4 Conclusions

This study systematically evaluated the quality differences between CAH and NAH by integrating physicochemical analysis, component detection, bioactivity assays, and network pharmacology. The results demonstrated significant discrepancies between CAH and NAH in key quality indicators, including total flavonoids, total polyphenols, amylase activity, proline content, and immunological activity, with some commercial samples failing to meet established standards. These findings highlight limitations in the current quality control system for acacia honey.

A total of 50 compounds were identified using SPE‐UPLC‐QE‐MS, and two flavonoids (hesperetin and pinocembrin) were screened as characteristic markers through chemometric and machine learning approaches. Importantly, these markers were experimentally validated to exhibit both immunomodulatory and anti‐inflammatory activities, supporting their dual role in reflecting compositional authenticity and biological functionality.

Mechanistically, network pharmacology and molecular docking analyses suggested that these markers may regulate multiple targets and pathways related to inflammation and immune response, with the PI3K–Akt pathway and MMP2 identified as key nodes. This provides a systems‐level perspective on their potential mode of action.

The characteristic markers of acacia honey identified in this research can compensate for the shortcomings of the current honey quality control system, offering critical technical backing for the precise authentication, quality supervision, and process optimization of high‐quality natural acacia honey. It specifically resolves the problem of uneven quality in commercially sold acacia honey and contributes to regulating the honey market order. The elucidated immunomodulatory and anti‐inflammatory mechanisms of the markers also provide a theoretical foundation for in‐depth exploration of acacia honey's nutritional and health values.

However, several limitations should be acknowledged. First, the bioactivity of the identified markers was validated only in vitro, lacking in vivo confirmation. Second, the molecular mechanisms were inferred primarily from network pharmacology and docking analyses, which require further experimental validation. Third, the sample size and geographical coverage of commercial honey may limit the generalizability of the findings.

Future studies should focus on in vivo validation, detailed mechanistic investigations at the molecular level, and the expansion of sample sources to establish more robust and universally applicable quality evaluation standards. In addition, the practical application of these markers in routine quality control and industrial processing warrants further exploration.

## Author Contributions

**Min Hua:** methodology, investigation, formal analysis, supervision. **Meng Li:** writing – review and editing, methodology, investigation, formal analysis, supervision. **Yuecheng Liu:** methodology, investigation, formal analysis, supervision. **Hongfu Sun:** writing – review and editing, visualization, supervision. **Yi Wu:** writing – review and editing, supervision, visualization. **Qi Gao:** writing – review and editing, visualization, supervision. **Yanpeng Dai:** writing – review and editing, methodology, supervision, project administration. **Qian Zhou:** writing – review and editing, resources, project administration, conceptualization, funding acquisition, supervision. **Meng Zhou:** methodology, supervision, writing – review and editing, project administration. **Bingkang Wang:** software, methodology, investigation, validation, formal analysis, visualization, writing – original draft, data curation. **Ning Zhang:** methodology, visualization, investigation.

## Funding

This work was supported by the National Natural Science Foundation of China, No. 81603299. Jinan “New 20 Policies” Funding Project for Universities, 202333089, 202228026. Shandong Provincial Natural Science Foundation, ZR2023LZY001. Shandong Provincial Traditional Chinese Medicine Science & Technology, M20252212.

## Conflicts of Interest

The authors declare no conflicts of interest.

## Supporting information


**Figure S1:** Dendrogram of HCA for honeys.
**Figure S2:** Permutation test.
**Figure S3:** The VIP plot by OPLS‐DA corresponding. N, NAH; C, CAH.
**Figure S4:** Venn diagram showing the intersection of chemical markers and inflammation targets.
**Figure S5:** Venn diagram showing the intersection of chemical markers and enhance immunity targets.
**Figure S6:** The “drug‐ingredient‐target‐disease” network diagram of chemical markers and anti‐inflammatory effects via cytoscape. Red: markers, purple: diseases, yellow: targets, blue: mechanism pathways.
**Figure S7:** The “drug‐ingredient‐target‐disease” network diagram of chemical markers and enhance immunity effects via cytoscape. Red: markers, purple: diseases, yellow: targets, blue: mechanism pathways.
**Table S1:** VIP values.
**Table S2:** Hesperetin targets.
**Table S3:** Pinocembrin targets.
**Table S4:** Swiss target prediction.

## Data Availability

The data that support the findings of this study are openly available in DAVID database at https://david.ncifcrf.gov.

## References

[fsn372021-bib-0001] Ahmad, N. , M. Y. Ansari , and T. M. Haqqi . 2020. “Role of iNOS in Osteoarthritis: Pathological and Therapeutic Aspects.” Journal of Cellular Physiology 235: 6366–6376. 10.1002/jcp.29607. 32017079 PMC8404685

[fsn372021-bib-0002] Akanda, M. K. M. , S. Mehjabin , and G. M. M. Parvez . 2024. “Honey for Nutrition and Health Benefits: An Overview.” In Honey Food Science Physiology, edited by R. Kumar , Y. A. Hajam , S. Bala Dhull , and A. Giri , 33–56. Springer Nature Singapore.

[fsn372021-bib-0003] An, Z. X. , T. Ye , J. W. Yu , et al. 2025. “A Novel Concoction Method of Chinese Medicinal and Edible Plants: Probiotic Fermentation, Sensory and Functional Composition Analysis.” Sustainable Food Technology 3, no. 3: 822–836. 10.1039/d5fb00091b.

[fsn372021-bib-0004] Biluca, F. C. , B. da Silva , T. Caon , et al. 2020. “Investigation of Phenolic Compounds, Antioxidant and Anti‐Inflammatory Activities in Stingless Bee Honey (Meliponinae).” Food Research International 129: 108756. 10.1016/j.foodres.2019.108756. 32036884

[fsn372021-bib-0005] Council Directive 2001/110/EC of 20 December 2001 Relating to Honey . 2001.

[fsn372021-bib-0006] Cucu, A.‐A. , G.‐M. Baci , A. R. Moise , et al. 2021. “Towards a Better Understanding of Nutritional and Therapeutic Effects of Honey and Their Applications in Apitherapy.” Applied Sciences 11: 4190. 10.3390/app11094190.

[fsn372021-bib-0007] Cucu, A.‐A. , O. Bobiș , V. Bonta , et al. 2024. “Unraveling the Physicochemical, Nutritional and Antioxidant Properties of the Honey Produced From the *Fallopia japonica* Plant.” Food 13: 1959. 10.3390/foods13131959. PMC11240986 38998468

[fsn372021-bib-0008] da Silva, P. M. , C. Gauche , L. V. Gonzaga , A. C. O. Costa , and R. Fett . 2016. “Honey: Chemical Composition, Stability and Authenticity.” Food Chemistry 196: 309–323. 10.1016/j.foodchem.2015.09.051. 26593496

[fsn372021-bib-0009] Damto, T. , A. Zewdu , and T. Birhanu . 2024. “Impact of Different Adulterants on Honey Quality Properties and Evaluating Different Analytical Approaches for Adulteration Detection.” Journal of Food Protection 87: 100241. 38360408 10.1016/j.jfp.2024.100241

[fsn372021-bib-0010] Deng, Q. , W. Chen , B. Deng , et al. 2024. “Based on Network Pharmacology, Molecular Docking and Experimental Verification to Reveal the Mechanism of *Andrographis paniculata* Against Solar Dermatitis.” Phytomedicine 135: 156025. 10.1016/j.phymed.2024.156025. 39326136

[fsn372021-bib-0041] EEC . 1986. “Council Directive 86/609/EEC of 24 November 1986 on the Approximation of Laws, Regulations and Administrative Provisions of the Member States Regarding the Protection of Animals Used for Experimental and Other Scientific Purposes.” Official Journal of the European Communities L358: 1–29.

[fsn372021-bib-0011] Escuredo, O. , I. Dobre , M. Fernández‐González , and M. C. Seijo . 2014. “Contribution of Botanical Origin and Sugar Composition of Honeys on the Crystallization Phenomenon.” Food Chemistry 149: 84–90. 10.1016/j.foodchem.2013.10.097. 24295680

[fsn372021-bib-0012] Ganesan, I. P. , and H. Kiyokawa . 2025. “A Perspective on Therapeutic Targeting Against Ubiquitin Ligases to Stabilize Tumor Suppressor Proteins.” Cancers 17: 626. 10.3390/cancers17040626. 40002221 PMC11853300

[fsn372021-bib-0013] GB 14963‐2011 National Food Safety Standard‐Honey . 2012.

[fsn372021-bib-0014] Ghramh, H. A. , E. H. Ibrahim , and Z. Ahmad . 2021. “Antimicrobial, Immunomodulatory and Cytotoxic Activities of Green Synthesized Nanoparticles From Acacia Honey and *Calotropis procera* .” Saudi Journal of Biological Sciences 28: 3367–3373. 10.1016/j.sjbs.2021.02.085. 34121874 PMC8175998

[fsn372021-bib-0015] Gonzalez Viejo, C. , D. D. Torrico , F. R. Dunshea , and S. Fuentes . 2019. “Development of Artificial Neural Network Models to Assess Beer Acceptability Based on Sensory Properties Using a Robotic Pourer: A Comparative Model Approach to Achieve an Artificial Intelligence System.” Beverages 5: 33. 10.3390/beverages5020033.

[fsn372021-bib-0016] Jordan, M. I. , and T. M. Mitchell . 2015. “Machine Learning: Trends, Perspectives, and Prospects.” Science 349: 255–260. 10.1126/science.aaa8415. 26185243

[fsn372021-bib-0017] Lawag, I. L. , E. S. Nolden , A. A. M. Schaper , L. Y. Lim , and C. Locher . 2023. “A Modified Folin‐Ciocalteu Assay for the Determination of Total Phenolics Content in Honey.” Applied Sciences 13, no. 4: 2135. 10.3390/app13042135.

[fsn372021-bib-0018] Li, J. , J. Zhang , Y. Liu , X. Yu , and X. Wang . 2023. “Simultaneous Determination of 26 Endogenous Components in Honey by Automated Solid Phase Extraction Coupled With Ultra‐High Performance Liquid Chromatography‐Tandem Mass Spectrometry.” Food Science 44: 265–272.

[fsn372021-bib-0019] Li, T. 2023. Optimization of Preparation Process for *Portulaca oleracea* L. Polysaccharide Liposomes and Preliminary Evaluation of its Immune Enhancement Activity. M.S. Thesis. Heilongjiang Bayi Agricultural University.

[fsn372021-bib-0020] Lin, B. , B. J. Daniels , M. J. Middleditch , et al. 2020. “Utility of the *Leptospermum scoparium* Compound Lepteridine as a Chemical Marker for Manuka Honey Authenticity.” ACS Omega 5: 8858–8866. 10.1021/acsomega.0c00486. 32337448 PMC7178798

[fsn372021-bib-0021] Liu, X. , N. Qi , H. Li , et al. 2025. “Main Quality and Characteristic Components Analysis of Huyou Honey.” Food Fermentation India 20: 1–11. 10.13995/j.cnki.11-1802/ts.041579.

[fsn372021-bib-0022] Liu, X. , J. Sun , P. Ji , et al. 2023. “Hydroxy Fatty Acids as Novel Markers for Authenticity Identification of the Honey Entomological Origin Based on the GC‐MS Method.” Journal of Agricultural and Food Chemistry 71: 7163–7173. 10.1021/acs.jafc.3c00835. 37096970

[fsn372021-bib-0023] Loh, Y. Y. , J. Anantharajan , Q. Huang , et al. 2024. “Identification of Small‐Molecule Binding Sites of a Ubiquitin‐Conjugating Enzyme‐UBE2T Through Fragment‐Based Screening.” Protein Science 33, no. 3: e4904. 10.1002/pro.4904. 38358126 PMC10868430

[fsn372021-bib-0024] Luca, L. , D. Pauliuc , F. Ursachi , and M. Oroian . 2025. “Physicochemical Parameters, Microbiological Quality, and Antibacterial Activity of Honey From the Bucovina Region of Romania.” Scientific Reports 15: 4358. 10.1038/s41598-025-88613-0. 39910223 PMC11799142

[fsn372021-bib-0025] Majewska, E. , B. Drużyńska , and R. Wołosiak . 2019. “Determination of the Botanical Origin of Honeybee Honeys Based on the Analysis of Their Selected Physicochemical Parameters Coupled With Chemometric Assays.” Food Science and Biotechnology 28: 1307–1314. 10.1007/s10068-019-00598-5. 31695929 PMC6811459

[fsn372021-bib-0026] Navia, S. H. , L. Vega , T. Rodríguez , and M. Rodríguez‐Sosa . 2026. “Immunomodulatory Effects of Flavonoids in Colitis‐Associated Colorectal Cancer.” International Journal of Molecular Sciences 27, no. 4: 1883. 10.3390/ijms27041883. 41752019 PMC12941120

[fsn372021-bib-0027] Nie, W. , S. Alimujiang , Y. Zhang , S. Zhang , and W. Li . 2025. “A Multi‐Omics Approach Combining GC‐MS, LC‐MS, and FT‐NIR With Chemometrics and Machine Learning for Metabolites Systematic Profiling and Geographical Origin Tracing of Artemisia Argyi Folium.” Journal of Chromatography. A 1757: 466138. 10.1016/j.chroma.2025.466138. 40527046

[fsn372021-bib-0028] Pantsar, T. , and A. Poso . 2018. “Binding Affinity via Docking: Fact and Fiction.” Molecules 23, no. 8: 81899. 10.3390/molecules23081899. PMC6222344 30061498

[fsn372021-bib-0029] Qi, N. , W. Zhao , C. Xue , et al. 2025. “Phenolic Acid and Flavonoid Content Analysis With Antioxidant Activity Assessment in Chinese C. Pi. Shen Honey.” Molecules 30: 370. 10.3390/molecules30020370. 39860240 PMC11767644

[fsn372021-bib-0030] Ren, C. , K. Wang , T. Luo , et al. 2022. “Kaempferol‐3‐*O*‐Galactoside as a Marker for Authenticating *Lespedeza bicolor* Turcz. Monofloral Honey.” Food Research International 160: 111667. 10.1016/j.foodres.2022.111667. 36076382

[fsn372021-bib-0032] Sun, J. 2022. The Difference of Components Between Maturehoney and Immature Honey by Metabolomics. M.S. Thesis. North West China University.

[fsn372021-bib-0033] Sun, J. , H. Zhao , F. Wu , et al. 2021. “Molecular Mechanism of Mature Honey Formation by GC‐MS‐ and LC‐MS‐Based Metabolomics.” Journal of Agricultural and Food Chemistry 69: 3362–3370. 10.1021/acs.jafc.1c00318. 33688728

[fsn372021-bib-0031] Sun, Z. 2017. The analysis and application of amino acids in honey. M.S. Thesis. North West China University.

[fsn372021-bib-0034] Wang, F. , J. Lou , X. Gao , et al. 2023. “Spleen‐Targeted Nanosystems for Immunomodulation.” Nano Today 52: 101943. 10.1016/j.nantod.2023.101943.

[fsn372021-bib-0035] Wei, Y. , S. Chen , Y. Ling , W. Wang , and Y. Huang . 2025. “Multi‐Omics Revealed That the Postbiotic of Hawthorn‐Probiotic Alleviated Constipation Caused by Loperamide in Elderly Mice.” Frontiers in Nutrition 12: 1498004. 10.3389/fnut.2025.1498004. 40070478 PMC11895004

[fsn372021-bib-0036] Wu, J. , X.‐T. Xu , C. Xing , et al. 2025. “Metabolic Profiling and Evaluation of Antioxidant and Anti‐Inflammatory Properties of * Apis cerana Cerana* Honey From Sansha City, Hainan Province, China.” Food Chemistry 475: 143256. 10.1016/j.foodchem.2025.143256. 39938270

[fsn372021-bib-0037] Zhang, H. , S. Kim , and W. Im . 2022. “Practical Guidance for Consensus Scoring and Force Field Selection in Protein–Ligand Binding Free Energy Simulations.” Journal of Chemical Information and Modeling 62, no. 23: 6084–6093. 10.1021/acs.jcim.2c01115. 36399655 PMC9772090

[fsn372021-bib-0038] Zhang, R. , H. Tian , S. Su , J. Zhu , Y. Yang , and W. Liu . 2017. “Comprehensive Evaluation Study of Honey Quality Based on Endogenous Components.” Food and Fermentation Industries Editorial Staff 43: 237–240. 10.13995/j.cnki.11-1802/ts.201701039.

[fsn372021-bib-0039] Zhang, Y. , X. Fu , L. Zhang , Q. Zhou , and W. Wang . 2025. “Splenic Volume as a Predictor of Survival in Cancer Patients Treated With Immune Checkpoint Inhibitors.” Frontiers in Immunology 16: 1598484. 10.3389/fimmu.2025.1598484. 40519921 PMC12163055

[fsn372021-bib-0040] Živkov Baloš, M. , N. Popov , S. Jakšić , et al. 2023. “Sunflower Honey‐Evaluation of Quality and Stability During Storage.” Foods Basel Switzerland 12: 2585. 10.3390/foods12132585. 37444323 PMC10340359

