# Supplementary material for: Two Flavones as Markers for the Distinction of Natural Acacia Honey Based on the UHPLC‐QE‐MS Method and Research on Their Potential Immunomodulatory Activity
Source: Food Sci Nutr. 2026 Jun 17;14(6):e72021. doi: 10.1002/fsn3.72021 (PMC13273532; doi:10.1002/fsn3.72021)
Supplement: Supplementary file 1 — Figure S1: Dendrogram of HCA for honeys. Figure S2: Permutation test. Figure S3: The VIP plot by OPLS‐DA corresponding. N, NAH; C, CAH. Figure S4: Venn diagram showing the intersection of chemical markers and inflammation targets. Figure S5: Venn diagram showing the intersection of chemical markers and enhance immunity targets. Figure S6: The “drug‐ingredient‐target‐disease” network diagram of chemical markers and anti‐inflammatory effects via cytoscape. Red: markers, purple: diseases, yellow: targets, blue: mechanism pathways. Figure S7: The “drug‐ingredient‐target‐disease” network diagram of chemical markers and enhance immunity effects via cytoscape. Red: markers, purple: diseases, yellow: targets, blue: mechanism pathways. Table S1: VIP values. Table S2: Hesperetin targets. Table S3: Pinocembrin targets. Table S4: Swiss target prediction. [file FSN3-14-e72021-s001.docx]

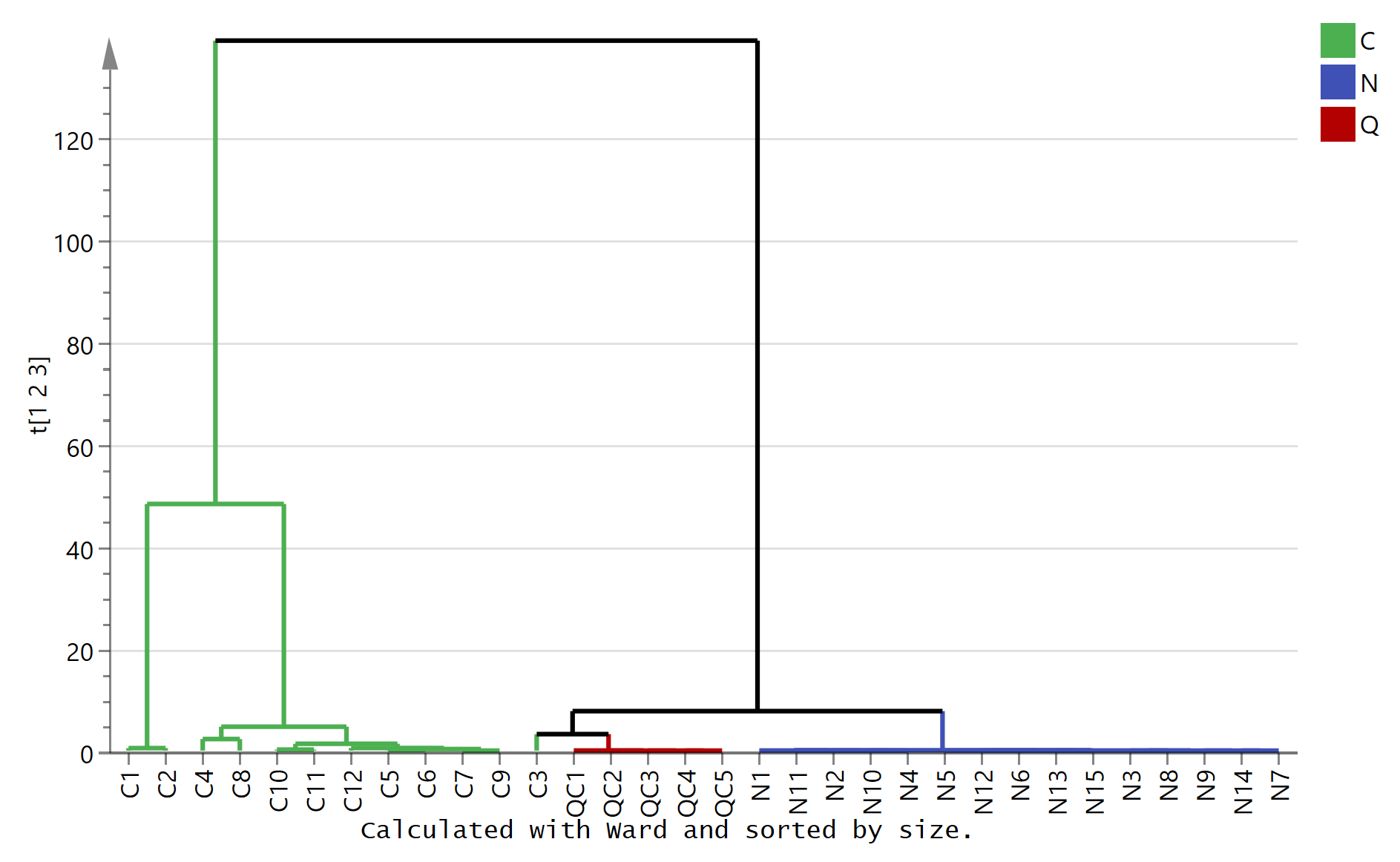


Figure S1 Dendrogram of HCA for honeys


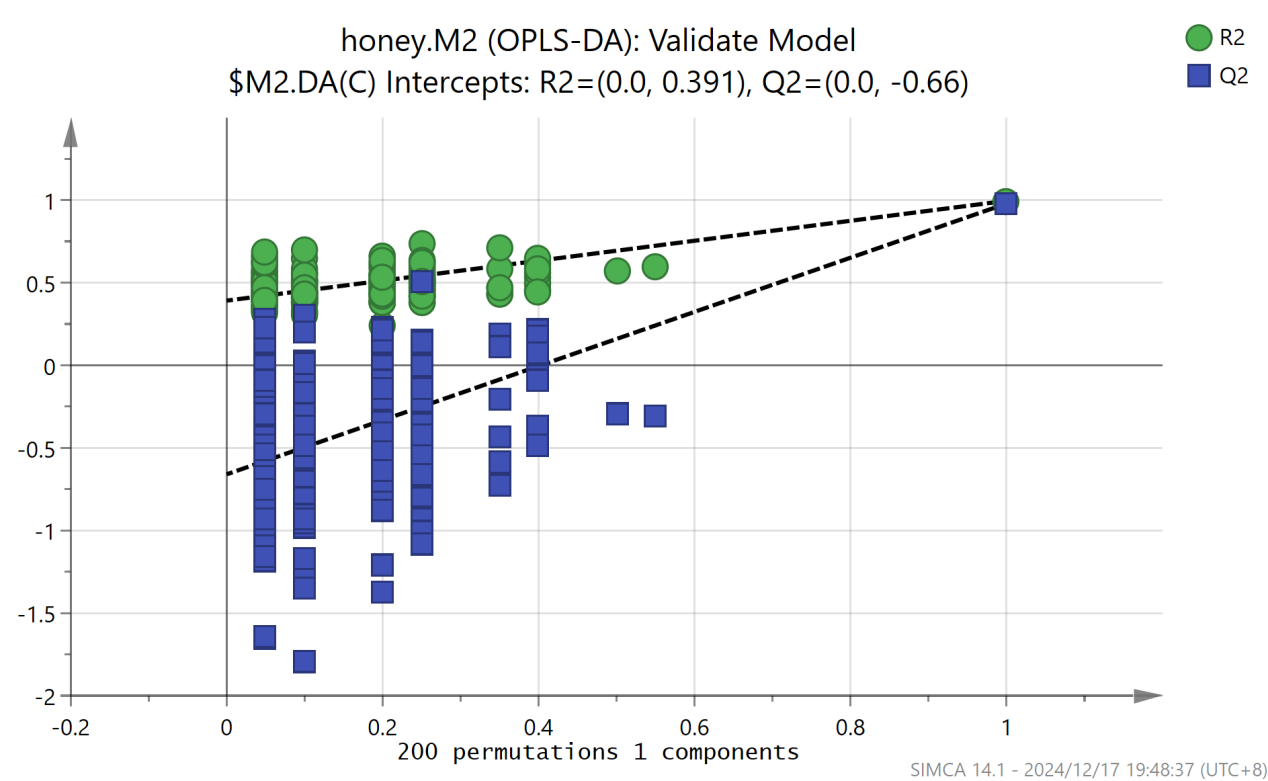


Figure S2 Permutation test


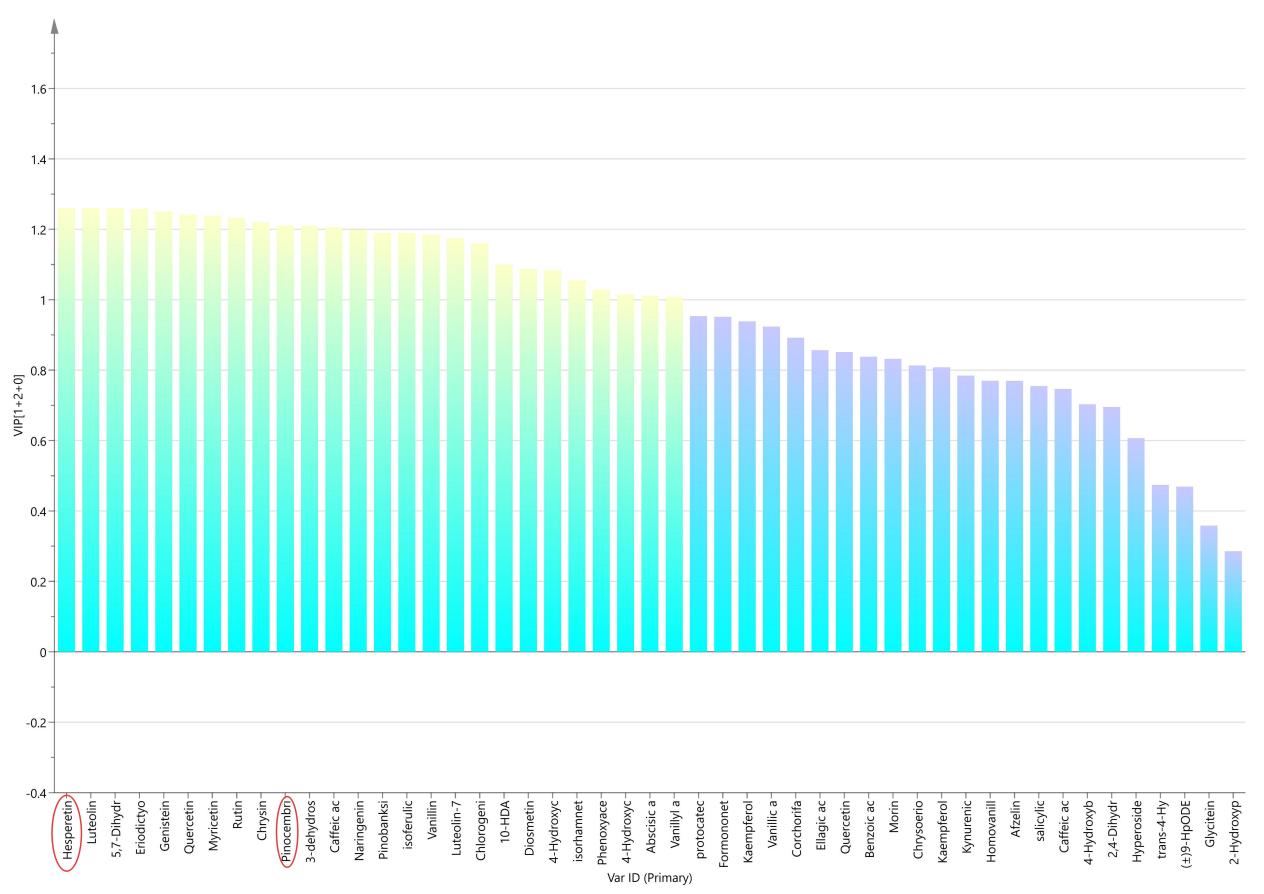


Figure S3 The VIP plot by OPLS-DA corresponding. N: NAH; C: CAH


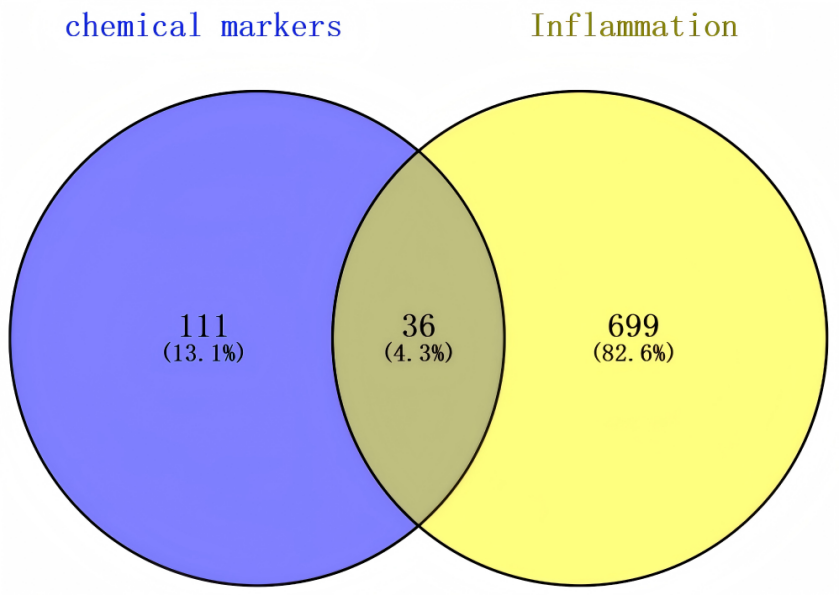


Figure S4 Venn diagram showing the intersection of chemical markers and inflammation targets


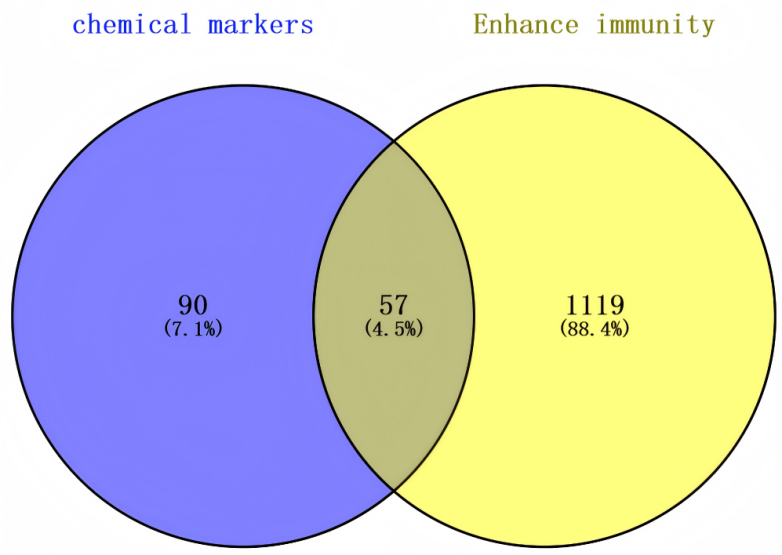


Figure S5 Venn diagram showing the intersection of chemical markers and enhance immunity targets


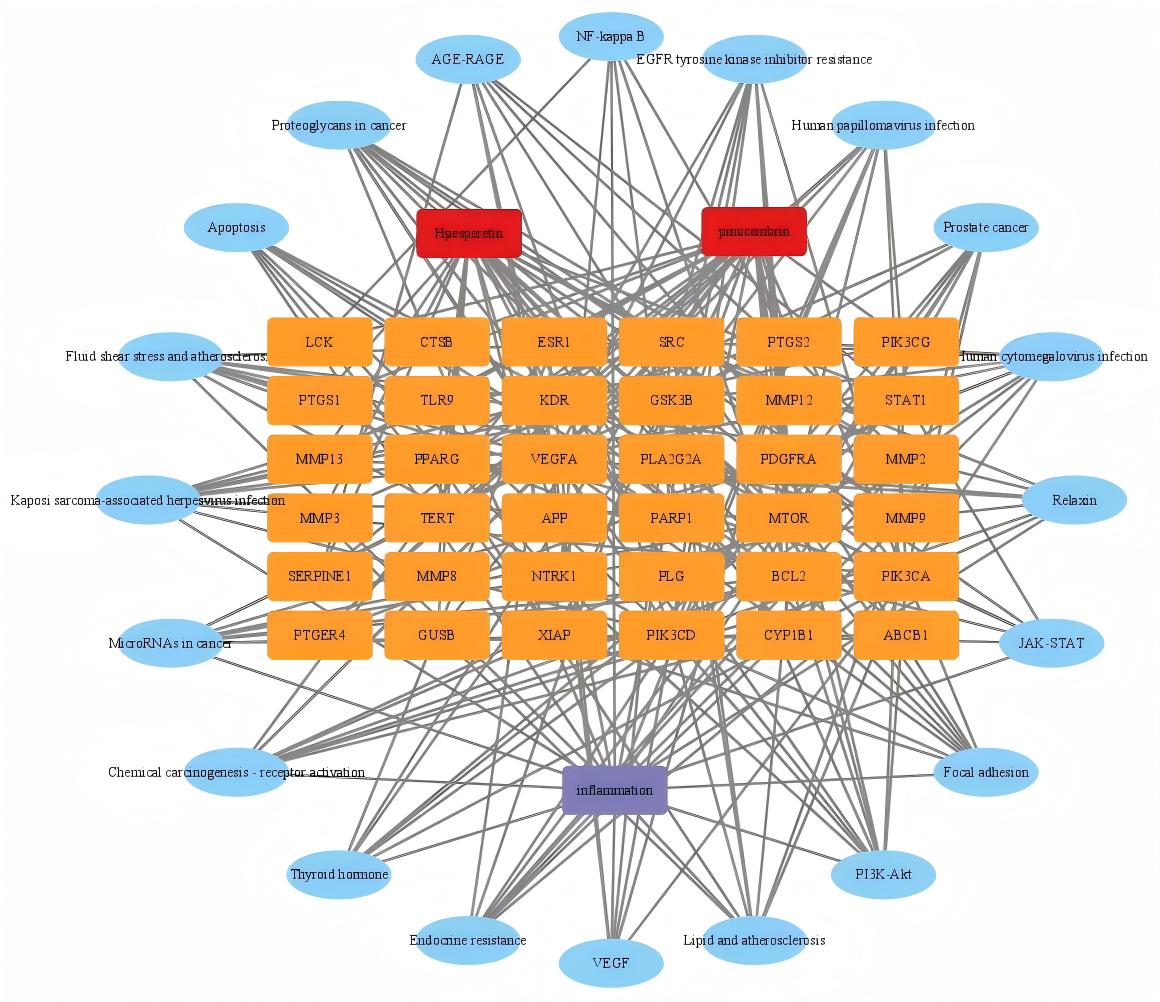


Figure S6 The "drug-ingredient-target-disease" network diagram of chemical markers and anti-inflammatory effects via cytoscape. Red: markers, purple: diseases, yellow: targets, blue: mechanism pathways


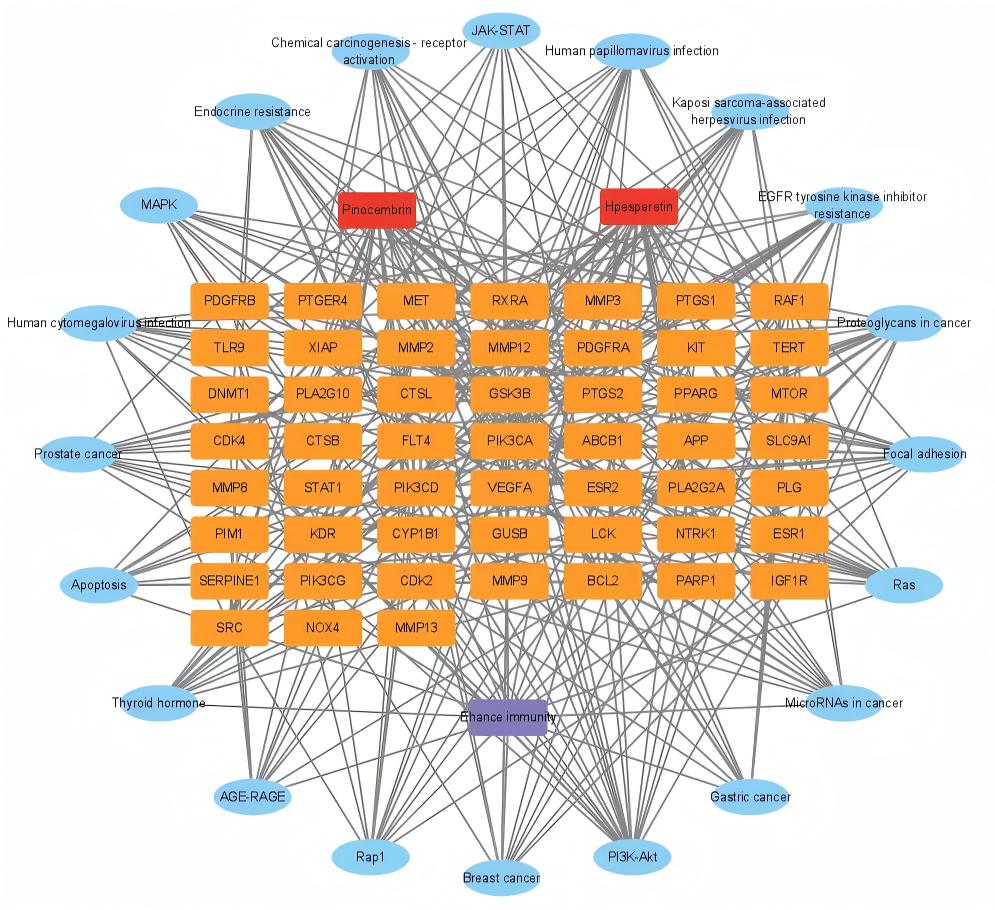


Figure S7 The "drug-ingredient-target-disease" network diagram of chemical markers and enhance immunity effects via cytoscape. Red: markers, purple: diseases, yellow: targets, blue: mechanism pathways

Table S1 VIP values

| Var ID (Primary) | M2.VIP[1+2+0] |
| --- | --- |
| Hesperetin | 1.25948 |
| Luteolin | 1.25926 |
| 5,7-Dihydroxy-2-(3-hydroxy-4-methoxyphenyl)chroman-4-one | 1.25921 |
| Eriodictyol | 1.25771 |
| Genistein | 1.25005 |
| Quercetin 3,4'-diglucoside | 1.24167 |
| Myricetin | 1.23803 |
| Rutin | 1.23232 |
| Chrysin | 1.21998 |
| Pinocembrin | 1.21066 |
| 3-dehydroshikimic acid | 1.20974 |
| Caffeic acid phenethyl ester | 1.20559 |
| Naringenin | 1.1983 |
| Pinobanksin | 1.18927 |
| isoferulic acid | 1.18885 |
| Vanillin | 1.18524 |
| Luteolin-7-O-glucoside | 1.17495 |
| Chlorogenic acid | 1.15946 |
| 10-HDA | 1.09978 |
| Diosmetin | 1.08761 |
| 4-Hydroxycinnamic acid | 1.08351 |
| isorhamnetin | 1.05446 |
| Phenoxyacetic acid | 1.02807 |
| 4-Hydroxycoumarin | 1.01489 |
| Abscisic acid | 1.01079 |
| Vanillyl alcohol | 1.00822 |
| protocatechuic acid | 0.95322 |
| Formononetin | 0.951161 |
| Kaempferol | 0.938657 |
| Vanillic acid | 0.923513 |
| Corchorifatty acid F | 0.891958 |
| Ellagic acid | 0.856396 |
| Quercetin | 0.851551 |
| Benzoic acid | 0.838103 |
| Morin | 0.831904 |
| Chrysoeriol | 0.812984 |
| Kaempferol-3-0-rutinoside（Nicotiflorin） | 0.808024 |
| Kynurenic acid | 0.784331 |
| Homovanillic acid | 0.76993 |
| Afzelin | 0.769585 |
| salicylic acid | 0.754787 |
| Caffeic acid | 0.746333 |
| 4-Hydroxybenzoic acid | 0.702752 |
| 2,4-Dihydroxybenzoic acid | 0.695056 |
| Hyperoside | 0.606686 |
| trans-4-Hydroxycinnamic acid(P-香豆酸) | 0.474322 |
| (±)9-HpODE | 0.469196 |
| Glycitein | 0.358519 |
| 2-Hydroxyphenylacetic acid | 0.285854 |

Table S2 Hesperetin Targets

| **Target** | **Common name** | **Uniprot ID** | **ChEMBL ID** | **Target Class** | **Probability*** | **Known actives**  **(3D/2D)** |
| --- | --- | --- | --- | --- | --- | --- |
| Carbonic anhydrase VII | CA7 | P43166 | CHEMBL2326 | Lyase | 1 | 11 / 8 |
| Carbonic anhydrase XII | CA12 | O43570 | CHEMBL3242 | Lyase | 1 | 45 / 7 |
| Carbonic anhydrase IV | CA4 | P22748 | CHEMBL3729 | Lyase | 1 | 11 / 8 |
| Cytochrome P450 1B1 | CYP1B1 | Q16678 | CHEMBL4878 | Cytochrome P450 | 1 | 4 / 6 |
| Cytochrome P450 19A1 | CYP19A1 | P11511 | CHEMBL1978 | Cytochrome P450 | 0.206265233 | 15 / 50 |
| Taste receptor type 2 member 31 | TAS2R31 | P59538 | CHEMBL2034804 | Taste family G protein-coupled receptor | 0.166798772 | 3 / 3 |
| Adenosine A1 receptor (by homology) | ADORA1 | P30542 | CHEMBL226 | Family A G protein-coupled receptor | 0.166798772 | 24 / 3 |
| Adenosine A3 receptor | ADORA3 | P0DMS8 | CHEMBL256 | Family A G protein-coupled receptor | 0.166798772 | 11 / 3 |
| ATP-binding cassette sub-family G member 2 | ABCG2 | Q9UNQ0 | CHEMBL5393 | Primary active transporter | 0.158886034 | 15 / 11 |
| Estradiol 17-beta-dehydrogenase 1 | HSD17B1 | P14061 | CHEMBL3181 | Enzyme | 0.158886034 | 48 / 1 |
| Estrogen receptor beta | ESR2 | Q92731 | CHEMBL242 | Nuclear receptor | 0.15098181 | 87 / 79 |
| Estrogen receptor alpha | ESR1 | P03372 | CHEMBL206 | Nuclear receptor | 0.135202128 | 92 / 87 |
| Monoamine oxidase B | MAOB | P27338 | CHEMBL2039 | Oxidoreductase | 0.135202128 | 25 / 122 |
| Multidrug resistance-associated protein 1 | ABCC1 | P33527 | CHEMBL3004 | Primary active transporter | 0.12730257 | 1 / 6 |
| Testis-specific androgen-binding protein | SHBG | P04278 | CHEMBL3305 | Secreted protein | 0.12730257 | 3 / 3 |
| Carbonyl reductase [NADPH] 1 | CBR1 | P16152 | CHEMBL5586 | Enzyme | 0.12730257 | 1 / 1 |
| Matrix metalloproteinase 13 | MMP13 | P45452 | CHEMBL280 | Protease | 0.12730257 | 96 / 1 |
| Cyclooxygenase-1 | PTGS1 | P23219 | CHEMBL221 | Oxidoreductase | 0.119403562 | 12 / 13 |
| Beta-secretase 1 | BACE1 | P56817 | CHEMBL4822 | Protease | 0.111501865 | 169 / 15 |
| Matrix metalloproteinase 12 | MMP12 | P39900 | CHEMBL4393 | Protease | 0.111501865 | 12 / 3 |
| Metabotropic glutamate receptor 5 | GRM5 | P41594 | CHEMBL3227 | Family C G protein-coupled receptor | 0.111501865 | 0 / 10 |
| Tyrosine-protein kinase SRC | SRC | P12931 | CHEMBL267 | Kinase | 0.111501865 | 26 / 4 |
| Aldo-keto-reductase family 1 member C3 | AKR1C3 | P42330 | CHEMBL4681 | Enzyme | 0.111501865 | 0 / 18 |
| Phospholipase A2 group 1B | PLA2G1B | P04054 | CHEMBL4426 | Enzyme | 0.111501865 | 0 / 1 |
| Kallikrein 1 | KLK1 | P06870 | CHEMBL2319 | Protease | 0.111501865 | 0 / 1 |
| Kallikrein 2 | KLK2 | P20151 | CHEMBL2442 | Protease | 0.111501865 | 0 / 1 |
| Carbonic anhydrase III | CA3 | P07451 | CHEMBL2885 | Lyase | 0.111501865 | 0 / 2 |
| Neuronal acetylcholine receptor protein alpha-7 subunit | CHRNA7 | P36544 | CHEMBL2492 | Ligand-gated ion channel | 0.111501865 | 10 / 0 |
| Telomerase reverse transcriptase | TERT | O14746 | CHEMBL2916 | Enzyme | 0.111501865 | 5 / 3 |
| Carbonic anhydrase II | CA2 | P00918 | CHEMBL205 | Lyase | 0.111501865 | 73 / 4 |
| Carbonic anhydrase I | CA1 | P00915 | CHEMBL261 | Lyase | 0.111501865 | 67 / 3 |
| Carbonic anhydrase VI | CA6 | P23280 | CHEMBL3025 | Lyase | 0.111501865 | 4 / 2 |
| Carbonic anhydrase VA | CA5A | P35218 | CHEMBL4789 | Lyase | 0.111501865 | 5 / 3 |
| DNA polymerase beta (by homology) | POLB | P06746 | CHEMBL2392 | Enzyme | 0.111501865 | 0 / 3 |
| Plasminogen activator inhibitor-1 | SERPINE1 | P05121 | CHEMBL3475 | Secreted protein | 0.111501865 | 2 / 4 |
| Beta amyloid A4 protein | APP | P05067 | CHEMBL2487 | Membrane receptor | 0.111501865 | 13 / 3 |
| Phospholipase A2 group V | PLA2G5 | P39877 | CHEMBL4323 | Enzyme | 0.111501865 | 0 / 3 |
| Group X secretory phospholipase A2 | PLA2G10 | O15496 | CHEMBL4342 | Enzyme | 0.111501865 | 0 / 1 |
| Plasminogen | PLG | P00747 | CHEMBL1801 | Protease | 0.111501865 | 2 / 0 |
| NADPH oxidase 4 | NOX4 | Q9NPH5 | CHEMBL1250375 | Enzyme | 0.111501865 | 8 / 0 |
| Carbonic anhydrase XIII | CA13 | Q8N1Q1 | CHEMBL3912 | Lyase | 0.111501865 | 2 / 2 |
| Carbonic anhydrase VB | CA5B | Q9Y2D0 | CHEMBL3969 | Lyase | 0.111501865 | 3 / 2 |
| Acetylcholinesterase | ACHE | P22303 | CHEMBL220 | Hydrolase | 0.111501865 | 10 / 50 |
| Beta-glucuronidase | GUSB | P08236 | CHEMBL2728 | Enzyme | 0.111501865 | 8 / 0 |
| Acyl coenzyme A:cholesterol acyltransferase | CES1 | P23141 | CHEMBL2265 | Enzyme | 0.111501865 | 2 / 1 |
| Sodium/glucose cotransporter 2 | SLC5A2 | P31639 | CHEMBL3884 | Electrochemical transporter | 0.111501865 | 2 / 3 |
| Carboxylesterase 2 | CES2 | O00748 | CHEMBL3180 | Enzyme | 0.111501865 | 1 / 1 |
| Retinoid X receptor alpha | RXRA | P19793 | CHEMBL2061 | Nuclear receptor | 0.111501865 | 0 / 18 |
| Vascular endothelial growth factor receptor 2 | KDR | P35968 | CHEMBL279 | Kinase | 0.111501865 | 63 / 12 |
| Metabotropic glutamate receptor 2 (by homology) | GRM2 | Q14416 | CHEMBL5137 | Family C G protein-coupled receptor | 0.111501865 | 0 / 72 |
| Aldose reductase | AKR1B1 | P15121 | CHEMBL1900 | Enzyme | 0.111501865 | 59 / 43 |
| Cyclin-dependent kinase 1/cyclin B1 | CDK1 CCNB1 | P06493 P14635 | CHEMBL1907602 | Other cytosolic protein | 0.111501865 | 5 / 0 |
| Cyclin-dependent kinase 2/cyclin E1 | CCNE1 CDK2 | P24864 P24941 | CHEMBL1907605 | Kinase | 0.111501865 | 10 / 0 |
| Dual specificity protein phosphatase 3 | DUSP3 | P51452 | CHEMBL2635 | Phosphatase | 0.111501865 | 3 / 0 |
| Nerve growth factor receptor Trk-A | NTRK1 | P04629 | CHEMBL2815 | Kinase | 0.111501865 | 3 / 0 |
| Serine/threonine-protein kinase Aurora-A | AURKA | O14965 | CHEMBL4722 | Kinase | 0.111501865 | 33 / 0 |
| Carbonic anhydrase IX | CA9 | Q16790 | CHEMBL3594 | Lyase | 0.111501865 | 53 / 1 |
| DNA topoisomerase I | TOP1 | P11387 | CHEMBL1781 | Isomerase | 0.111501865 | 4 / 0 |
| DNA (cytosine-5)-methyltransferase 1 | DNMT1 | P26358 | CHEMBL1993 | Writer | 0.111501865 | 0 / 1 |
| 6-phosphogluconate dehydrogenase | PGD | P52209 | CHEMBL3404 | Enzyme | 0.111501865 | 0 / 4 |
| CMP-N-acetylneuraminate-beta-1,4-galactoside alpha-2,3-sialyltransferase | ST3GAL3 | Q11203 | CHEMBL3596076 | Transferase | 0.111501865 | 0 / 1 |
| Alpha-(1,3)-fucosyltransferase 7 | FUT7 | Q11130 | CHEMBL3596077 | Transferase | 0.111501865 | 0 / 1 |
| Fucosyltransferase 4 | FUT4 | P22083 | CHEMBL4996 | Enzyme | 0.111501865 | 0 / 1 |
| Signal transducer and activator of transcription 1-alpha/beta | STAT1 | P42224 | CHEMBL6101 | Transcription factor | 0.111501865 | 0 / 1 |
| Squalene monooxygenase (by homology) | SQLE | Q14534 | CHEMBL3592 | Enzyme | 0.111501865 | 0 / 3 |
| Free fatty acid receptor 1 | FFAR1 | O14842 | CHEMBL4422 | Family A G protein-coupled receptor | 0.111501865 | 0 / 129 |
| Serine/threonine-protein kinase PIM1 | PIM1 | P11309 | CHEMBL2147 | Kinase | 0.111501865 | 12 / 0 |
| Serine/threonine-protein kinase PIM2 | PIM2 | Q9P1W9 | CHEMBL4523 | Kinase | 0.111501865 | 6 / 0 |
| Serine/threonine-protein kinase PIM3 | PIM3 | Q86V86 | CHEMBL5407 | Kinase | 0.111501865 | 3 / 0 |
| Matrix metalloproteinase 2 | MMP2 | P08253 | CHEMBL333 | Protease | 0.111501865 | 145 / 8 |
| Insulin-like growth factor I receptor | IGF1R | P08069 | CHEMBL1957 | Kinase | 0.111501865 | 15 / 0 |
| Matrix metalloproteinase 9 | MMP9 | P14780 | CHEMBL321 | Protease | 0.111501865 | 167 / 1 |
| Ornithine decarboxylase | ODC1 | P11926 | CHEMBL1869 | Lyase | 0.111501865 | 0 / 5 |
| Poly [ADP-ribose] polymerase-1 | PARP1 | P09874 | CHEMBL3105 | Enzyme | 0.111501865 | 20 / 0 |
| Dynamin-1 | DNM1 | Q05193 | CHEMBL4958 | Enzyme | 0.111501865 | 6 / 0 |
| Tankyrase-2 | TNKS2 | Q9H2K2 | CHEMBL6154 | Enzyme | 0.111501865 | 4 / 0 |
| Tankyrase-1 | TNKS | O95271 | CHEMBL6164 | Enzyme | 0.111501865 | 4 / 0 |
| Hepatocyte growth factor receptor | MET | P08581 | CHEMBL3717 | Kinase | 0.111501865 | 56 / 5 |
| Dual-specificity tyrosine-phosphorylation regulated kinase 1A | DYRK1A | Q13627 | CHEMBL2292 | Kinase | 0.111501865 | 23 / 1 |
| P-glycoprotein 1 | ABCB1 | P08183 | CHEMBL4302 | Primary active transporter | 0.111501865 | 1 / 188 |
| Peroxisome proliferator-activated receptor gamma | PPARG | P37231 | CHEMBL235 | Nuclear receptor | 0.111501865 | 1 / 118 |
| Mitogen-activated protein kinase kinase kinase kinase 4 | MAP4K4 | O95819 | CHEMBL6166 | Kinase | 0.111501865 | 3 / 0 |
| Phospholipase A2 group IIA | PLA2G2A | P14555 | CHEMBL3474 | Enzyme | 0.111501865 | 1 / 8 |
| Cyclin-dependent kinase 2/cyclin A | CDK2 CCNA1 CCNA2 | P24941 P78396 P20248 | CHEMBL2094128 | Other cytosolic protein | 0.111501865 | 13 / 0 |
| Protein-arginine N-methyltransferase 1 | PRMT1 | Q99873 | CHEMBL5524 | Writer | 0.111501865 | 4 / 0 |
| Estradiol 17-beta-dehydrogenase 2 | HSD17B2 | P37059 | CHEMBL2789 | Enzyme | 0.111501865 | 44 / 0 |
| Endothelin receptor ET-A | EDNRA | P25101 | CHEMBL252 | Family A G protein-coupled receptor | 0.111501865 | 12 / 81 |
| Apoptosis regulator Bcl-2 | BCL2 | P10415 | CHEMBL4860 | Other ion channel | 0.111501865 | 6 / 10 |
| Serine/threonine-protein kinase/endoribonuclease IRE1 | ERN1 | O75460 | CHEMBL1163101 | Enzyme | 0 | 22 / 0 |
| Estradiol 17-beta-dehydrogenase 3 | HSD17B3 | P37058 | CHEMBL4234 | Enzyme | 0 | 5 / 0 |
| Serine/threonine-protein kinase Chk1 | CHEK1 | O14757 | CHEMBL4630 | Kinase | 0 | 39 / 0 |
| Matrix metalloproteinase 8 | MMP8 | P22894 | CHEMBL4588 | Protease | 0 | 74 / 0 |
| Arachidonate 12-lipoxygenase | ALOX12 | P18054 | CHEMBL3687 | Enzyme | 0 | 0 / 4 |
| Purine nucleoside phosphorylase (by homology) | PNP | P00491 | CHEMBL4338 | Enzyme | 0 | 4 / 0 |
| Platelet-derived growth factor receptor beta | PDGFRB | P09619 | CHEMBL1913 | Kinase | 0 | 8 / 0 |
| Matrix metalloproteinase 3 | MMP3 | P08254 | CHEMBL283 | Protease | 0 | 64 / 0 |
| Stem cell growth factor receptor | KIT | P10721 | CHEMBL1936 | Kinase | 0 | 2 / 3 |
| Potassium-transporting ATPase | ATP4B ATP4A | P51164 P20648 | CHEMBL2095173 | Primary active transporter | 0 | 1 / 0 |
| Serine/threonine-protein kinase RAF | RAF1 | P04049 | CHEMBL1906 | Kinase | 0 | 10 / 0 |
| MAP kinase-activated protein kinase 2 | MAPKAPK2 | P49137 | CHEMBL2208 | Kinase | 0 | 7 / 0 |

Table S3 Pinocembrin Targets

| Target | Common name | Uniprot ID | ChEMBL ID | Target Class | Probability* | Known actives  (3D/2D) |
| --- | --- | --- | --- | --- | --- | --- |
| Cytochrome P450 19A1 | CYP19A1 | P11511 | CHEMBL1978 | Cytochrome P450 | 0.995336512 | 78 / 52 |
| Cytochrome P450 1B1 | CYP1B1 | Q16678 | CHEMBL4878 | Cytochrome P450 | 0.995336512 | 3 / 6 |
| ATP-binding cassette sub-family G member 2 | ABCG2 | Q9UNQ0 | CHEMBL5393 | Primary active transporter | 0.504893512 | 15 / 7 |
| Monoamine oxidase B | MAOB | P27338 | CHEMBL2039 | Oxidoreductase | 0.465623633 | 164 / 127 |
| Multidrug resistance-associated protein 1 | ABCC1 | P33527 | CHEMBL3004 | Primary active transporter | 0.347652208 | 0 / 2 |
| Testis-specific androgen-binding protein | SHBG | P04278 | CHEMBL3305 | Secreted protein | 0.347652208 | 4 / 3 |
| Carbonic anhydrase VII | CA7 | P43166 | CHEMBL2326 | Lyase | 0.339889056 | 51 / 7 |
| Carbonic anhydrase XII | CA12 | O43570 | CHEMBL3242 | Lyase | 0.339889056 | 105 / 6 |
| Estradiol 17-beta-dehydrogenase 1 | HSD17B1 | P14061 | CHEMBL3181 | Enzyme | 0.332035538 | 29 / 1 |
| Carbonic anhydrase IV | CA4 | P22748 | CHEMBL3729 | Lyase | 0.332035538 | 9 / 7 |
| Estrogen receptor alpha | ESR1 | P03372 | CHEMBL206 | Nuclear receptor | 0.324173835 | 43 / 85 |
| Estrogen receptor beta | ESR2 | Q92731 | CHEMBL242 | Nuclear receptor | 0.28470536 | 43 / 77 |
| Taste receptor type 2 member 31 | TAS2R31 | P59538 | CHEMBL2034804 | Taste family G protein-coupled receptor | 0.28470536 | 0 / 3 |
| Adenosine A1 receptor (by homology) | ADORA1 | P30542 | CHEMBL226 | Family A G protein-coupled receptor | 0.214160248 | 44 / 3 |
| Adenosine A3 receptor | ADORA3 | P0DMS8 | CHEMBL256 | Family A G protein-coupled receptor | 0.214160248 | 12 / 2 |
| Carbonyl reductase [NADPH] 1 | CBR1 | P16152 | CHEMBL5586 | Enzyme | 0.182723414 | 1 / 1 |
| Cyclooxygenase-1 | PTGS1 | P23219 | CHEMBL221 | Oxidoreductase | 0.174862805 | 11 / 12 |
| Phospholipase A2 group 1B | PLA2G1B | P04054 | CHEMBL4426 | Enzyme | 0.127750341 | 0 / 1 |
| Metabotropic glutamate receptor 5 | GRM5 | P41594 | CHEMBL3227 | Family C G protein-coupled receptor | 0.127750341 | 22 / 10 |
| Aldo-keto-reductase family 1 member C3 | AKR1C3 | P42330 | CHEMBL4681 | Enzyme | 0.119895127 | 2 / 7 |
| Carboxylesterase 2 | CES2 | O00748 | CHEMBL3180 | Enzyme | 0.119895127 | 4 / 1 |
| Beta-secretase 1 | BACE1 | P56817 | CHEMBL4822 | Protease | 0.119895127 | 36 / 14 |
| Acyl coenzyme A:cholesterol acyltransferase | CES1 | P23141 | CHEMBL2265 | Enzyme | 0.119895127 | 11 / 1 |
| Matrix metalloproteinase 13 | MMP13 | P45452 | CHEMBL280 | Protease | 0.119895127 | 109 / 2 |
| Matrix metalloproteinase 12 | MMP12 | P39900 | CHEMBL4393 | Protease | 0.119895127 | 9 / 3 |
| DNA polymerase beta (by homology) | POLB | P06746 | CHEMBL2392 | Enzyme | 0.112041901 | 0 / 2 |
| Phospholipase A2 group V | PLA2G5 | P39877 | CHEMBL4323 | Enzyme | 0.112041901 | 0 / 3 |
| Peroxisome proliferator-activated receptor gamma | PPARG | P37231 | CHEMBL235 | Nuclear receptor | 0.112041901 | 4 / 139 |
| Sodium/glucose cotransporter 2 | SLC5A2 | P31639 | CHEMBL3884 | Electrochemical transporter | 0.112041901 | 2 / 6 |
| Kallikrein 1 | KLK1 | P06870 | CHEMBL2319 | Protease | 0.112041901 | 0 / 1 |
| Kallikrein 2 | KLK2 | P20151 | CHEMBL2442 | Protease | 0.112041901 | 0 / 1 |
| Vascular endothelial growth factor receptor 2 | KDR | P35968 | CHEMBL279 | Kinase | 0.112041901 | 117 / 9 |
| Retinoid X receptor alpha | RXRA | P19793 | CHEMBL2061 | Nuclear receptor | 0.112041901 | 0 / 16 |
| Endothelin receptor ET-A | EDNRA | P25101 | CHEMBL252 | Family A G protein-coupled receptor | 0.112041901 | 4 / 26 |
| Butyrylcholinesterase | BCHE | P06276 | CHEMBL1914 | Hydrolase | 0.112041901 | 9 / 2 |
| Acetylcholinesterase | ACHE | P22303 | CHEMBL220 | Hydrolase | 0.112041901 | 19 / 21 |
| Phospholipase A2 group IIA | PLA2G2A | P14555 | CHEMBL3474 | Enzyme | 0.112041901 | 9 / 8 |
| Plasminogen activator inhibitor-1 | SERPINE1 | P05121 | CHEMBL3475 | Secreted protein | 0.112041901 | 1 / 3 |
| Group X secretory phospholipase A2 | PLA2G10 | O15496 | CHEMBL4342 | Enzyme | 0.112041901 | 3 / 1 |
| Hepatocyte growth factor receptor | MET | P08581 | CHEMBL3717 | Kinase | 0.112041901 | 95 / 4 |
| D-amino-acid oxidase | DAO | P14920 | CHEMBL5485 | Enzyme | 0.112041901 | 37 / 0 |
| Serine/threonine-protein kinase/endoribonuclease IRE1 | ERN1 | O75460 | CHEMBL1163101 | Enzyme | 0.112041901 | 64 / 0 |
| Vascular endothelial growth factor receptor 3 | FLT4 | P35916 | CHEMBL1955 | Kinase | 0.112041901 | 8 / 0 |
| Platelet-derived growth factor receptor alpha | PDGFRA | P16234 | CHEMBL2007 | Kinase | 0.112041901 | 9 / 0 |
| Serine/threonine-protein kinase Aurora-B | AURKB | Q96GD4 | CHEMBL2185 | Kinase | 0.112041901 | 38 / 0 |
| Glycogen synthase kinase-3 beta | GSK3B | P49841 | CHEMBL262 | Kinase | 0.112041901 | 119 / 0 |
| Serine/threonine-protein kinase Aurora-C | AURKC | Q9UQB9 | CHEMBL3935 | Kinase | 0.112041901 | 2 / 0 |
| Serine/threonine-protein kinase Aurora-A | AURKA | O14965 | CHEMBL4722 | Kinase | 0.112041901 | 73 / 0 |
| Tankyrase-2 | TNKS2 | Q9H2K2 | CHEMBL6154 | Enzyme | 0.112041901 | 9 / 0 |
| Tankyrase-1 | TNKS | O95271 | CHEMBL6164 | Enzyme | 0.112041901 | 12 / 0 |
| Aldose reductase | AKR1B1 | P15121 | CHEMBL1900 | Enzyme | 0.112041901 | 69 / 47 |
| Carbonic anhydrase III | CA3 | P07451 | CHEMBL2885 | Lyase | 0.112041901 | 0 / 3 |
| Platelet-derived growth factor receptor | PDGFRA PDGFRB | P16234 P09619 | CHEMBL2095189 | Kinase | 0.112041901 | 12 / 0 |
| Carbonic anhydrase II | CA2 | P00918 | CHEMBL205 | Lyase | 0.112041901 | 212 / 4 |
| Carbonic anhydrase I | CA1 | P00915 | CHEMBL261 | Lyase | 0.112041901 | 172 / 3 |
| Alkaline phosphatase placental-like | ALPG | P10696 | CHEMBL3402 | Enzyme | 0.112041901 | 6 / 0 |
| Dynamin-1 | DNM1 | Q05193 | CHEMBL4958 | Enzyme | 0.112041901 | 2 / 0 |
| Alkaline phosphatase, tissue-nonspecific isozyme | ALPL | P05186 | CHEMBL5979 | Enzyme | 0.112041901 | 24 / 0 |
| Carbonic anhydrase VI | CA6 | P23280 | CHEMBL3025 | Lyase | 0.112041901 | 6 / 2 |
| Monoamine oxidase A | MAOA | P21397 | CHEMBL1951 | Oxidoreductase | 0.112041901 | 97 / 22 |
| Inhibitor of apoptosis protein 3 | XIAP | P98170 | CHEMBL4198 | Other cytosolic protein | 0.112041901 | 2 / 0 |
| Beta-glucuronidase | GUSB | P08236 | CHEMBL2728 | Enzyme | 0.112041901 | 11 / 0 |
| Dual specificity phosphatase Cdc25A | CDC25A | P30304 | CHEMBL3775 | Phosphatase | 0.112041901 | 6 / 0 |
| ALK tyrosine kinase receptor | ALK | Q9UM73 | CHEMBL4247 | Kinase | 0.112041901 | 10 / 0 |
| Catechol O-methyltransferase | COMT | P21964 | CHEMBL2023 | Transferase | 0.112041901 | 12 / 0 |
| Ribosomal protein S6 kinase 2 | RPS6KB2 | Q9UBS0 | CHEMBL3111 | Kinase | 0.112041901 | 1 / 0 |
| Calmodulin | CALM1 | P62158 | CHEMBL6093 | Unclassified protein | 0.112041901 | 1 / 0 |
| Dual specificity phosphatase Cdc25C | CDC25C | P30307 | CHEMBL2378 | Phosphatase | 0.112041901 | 1 / 0 |
| Dual specificity phosphatase Cdc25B | CDC25B | P30305 | CHEMBL4804 | Phosphatase | 0.112041901 | 7 / 0 |
| Prostanoid EP1 receptor | PTGER1 | P34995 | CHEMBL1811 | Family A G protein-coupled receptor | 0.112041901 | 0 / 14 |
| Prostanoid EP4 receptor | PTGER4 | P35408 | CHEMBL1836 | Family A G protein-coupled receptor | 0.112041901 | 0 / 22 |
| Tyrosine-protein kinase LCK | LCK | P06239 | CHEMBL258 | Kinase | 0.112041901 | 45 / 0 |
| Cathepsin L | CTSL | P07711 | CHEMBL3837 | Protease | 0.112041901 | 12 / 2 |
| Cathepsin (B and K) | CTSB | P07858 | CHEMBL4072 | Protease | 0.112041901 | 5 / 1 |
| Cyclin-dependent kinase 2 | CDK2 | P24941 | CHEMBL301 | Kinase | 0.112041901 | 61 / 0 |
| Cyclin-dependent kinase 4 | CDK4 | P11802 | CHEMBL331 | Kinase | 0.112041901 | 20 / 0 |
| Tyrosine-protein kinase SRC | SRC | P12931 | CHEMBL267 | Kinase | 0.112041901 | 71 / 3 |
| Serine/threonine-protein kinase Nek1 | NEK1 | Q96PY6 | CHEMBL5855 | Kinase | 0.112041901 | 19 / 0 |
| Serine/threonine-protein kinase mTOR | MTOR | P42345 | CHEMBL2842 | Kinase | 0.112041901 | 120 / 0 |
| PI3-kinase p110-gamma subunit | PIK3CG | P48736 | CHEMBL3267 | Enzyme | 0.112041901 | 102 / 0 |
| PI3-kinase p110-alpha subunit | PIK3CA | P42336 | CHEMBL4005 | Enzyme | 0.112041901 | 110 / 0 |
| Toll-like receptor (TLR7/TLR9) | TLR9 | Q9NR96 | CHEMBL5804 | Toll-like and Il-1 receptors | 0.112041901 | 15 / 0 |
| Cyclooxygenase-2 | PTGS2 | P35354 | CHEMBL230 | Oxidoreductase | 0.112041901 | 37 / 12 |
| Metabotropic glutamate receptor 4 | GRM4 | Q14833 | CHEMBL2736 | Family C G protein-coupled receptor | 0.112041901 | 5 / 0 |
| Serine/threonine-protein kinase PLK4 | PLK4 | O00444 | CHEMBL3788 | Kinase | 0.112041901 | 14 / 0 |
| Carbonic anhydrase IX | CA9 | Q16790 | CHEMBL3594 | Lyase | 0.112041901 | 129 / 1 |
| Metabotropic glutamate receptor 2 | GRM2 | Q14416 | CHEMBL5137 | Family C G protein-coupled receptor | 0.112041901 | 2 / 73 |
| Histone deacetylase 7 | HDAC7 | Q8WUI4 | CHEMBL2716 | Eraser | 0.112041901 | 18 / 0 |
| Cytochrome P450 11B2 | CYP11B2 | P19099 | CHEMBL2722 | Cytochrome P450 | 0.112041901 | 20 / 0 |
| Lysine-specific histone demethylase 1 | KDM1A | O60341 | CHEMBL6136 | Eraser | 0.112041901 | 5 / 0 |
| Cyclin-dependent kinase 5/CDK5 activator 1 | CDK5R1 CDK5 | Q15078 Q00535 | CHEMBL1907600 | Kinase | 0.112041901 | 51 / 0 |
| Cyclin-dependent kinase 1/cyclin B | CCNB3 CDK1 CCNB1 CCNB2 | Q8WWL7 P06493 P14635 O95067 | CHEMBL2094127 | Other cytosolic protein | 0.112041901 | 24 / 0 |
| Hepatocyte nuclear factor 4-alpha | HNF4A | P41235 | CHEMBL5398 | Unclassified protein | 0.112041901 | 0 / 6 |
| Placenta growth factor | PGF | P49763 | CHEMBL1697671 | Unclassified protein | 0.112041901 | 0 / 3 |
| Vascular endothelial growth factor A | VEGFA | P15692 | CHEMBL1783 | Secreted protein | 0.112041901 | 0 / 3 |
| CDGSH iron-sulfur domain-containing protein 1 | CISD1 | Q9NZ45 | CHEMBL1795168 | Unclassified protein | 0.112041901 | 3 / 0 |
| Thymidylate synthase | TYMS | P04818 | CHEMBL1952 | Transferase | 0.112041901 | 28 / 0 |
| Sodium/hydrogen exchanger 1 | SLC9A1 | P19634 | CHEMBL2781 | Electrochemical transporter | 0.112041901 | 44 / 0 |
| PI3-kinase p110-delta subunit | PIK3CD | O00329 | CHEMBL3130 | Enzyme | 0.112041901 | 65 / 0 |
| DNA-dependent protein kinase | PRKDC | P78527 | CHEMBL3142 | Kinase | 0.112041901 | 41 / 0 |

Table S4 Swiss Target Prediction

| **Target** | **Common name** | **Uniprot ID** | **ChEMBL ID** | **Target Class** | **Probability*** | **Known actives**  **(3D/2D)** |
| --- | --- | --- | --- | --- | --- | --- |
| Carbonic anhydrase VII | CA7 | P43166 | CHEMBL2326 | Lyase | 1 | 11 / 8 |
| Carbonic anhydrase XII | CA12 | O43570 | CHEMBL3242 | Lyase | 1 | 45 / 7 |
| Carbonic anhydrase IV | CA4 | P22748 | CHEMBL3729 | Lyase | 1 | 11 / 8 |
| Cytochrome P450 1B1 | CYP1B1 | Q16678 | CHEMBL4878 | Cytochrome P450 | 1 | 4 / 6 |
| Cytochrome P450 19A1 | CYP19A1 | P11511 | CHEMBL1978 | Cytochrome P450 | 0.206265233 | 15 / 50 |
| Taste receptor type 2 member 31 | TAS2R31 | P59538 | CHEMBL2034804 | Taste family G protein-coupled receptor | 0.166798772 | 3 / 3 |
| Adenosine A1 receptor (by homology) | ADORA1 | P30542 | CHEMBL226 | Family A G protein-coupled receptor | 0.166798772 | 24 / 3 |
| Adenosine A3 receptor | ADORA3 | P0DMS8 | CHEMBL256 | Family A G protein-coupled receptor | 0.166798772 | 11 / 3 |
| ATP-binding cassette sub-family G member 2 | ABCG2 | Q9UNQ0 | CHEMBL5393 | Primary active transporter | 0.158886034 | 15 / 11 |
| Estradiol 17-beta-dehydrogenase 1 | HSD17B1 | P14061 | CHEMBL3181 | Enzyme | 0.158886034 | 48 / 1 |
| Estrogen receptor beta | ESR2 | Q92731 | CHEMBL242 | Nuclear receptor | 0.15098181 | 87 / 79 |
| Estrogen receptor alpha | ESR1 | P03372 | CHEMBL206 | Nuclear receptor | 0.135202128 | 92 / 87 |
| Monoamine oxidase B | MAOB | P27338 | CHEMBL2039 | Oxidoreductase | 0.135202128 | 25 / 122 |
| Multidrug resistance-associated protein 1 | ABCC1 | P33527 | CHEMBL3004 | Primary active transporter | 0.12730257 | 1 / 6 |
| Testis-specific androgen-binding protein | SHBG | P04278 | CHEMBL3305 | Secreted protein | 0.12730257 | 3 / 3 |
| Carbonyl reductase [NADPH] 1 | CBR1 | P16152 | CHEMBL5586 | Enzyme | 0.12730257 | 1 / 1 |
| Matrix metalloproteinase 13 | MMP13 | P45452 | CHEMBL280 | Protease | 0.12730257 | 96 / 1 |
| Cyclooxygenase-1 | PTGS1 | P23219 | CHEMBL221 | Oxidoreductase | 0.119403562 | 12 / 13 |
| Beta-secretase 1 | BACE1 | P56817 | CHEMBL4822 | Protease | 0.111501865 | 169 / 15 |
| Matrix metalloproteinase 12 | MMP12 | P39900 | CHEMBL4393 | Protease | 0.111501865 | 12 / 3 |
| Metabotropic glutamate receptor 5 | GRM5 | P41594 | CHEMBL3227 | Family C G protein-coupled receptor | 0.111501865 | 0 / 10 |
| Tyrosine-protein kinase SRC | SRC | P12931 | CHEMBL267 | Kinase | 0.111501865 | 26 / 4 |
| Aldo-keto-reductase family 1 member C3 | AKR1C3 | P42330 | CHEMBL4681 | Enzyme | 0.111501865 | 0 / 18 |
| Phospholipase A2 group 1B | PLA2G1B | P04054 | CHEMBL4426 | Enzyme | 0.111501865 | 0 / 1 |
| Kallikrein 1 | KLK1 | P06870 | CHEMBL2319 | Protease | 0.111501865 | 0 / 1 |
| Kallikrein 2 | KLK2 | P20151 | CHEMBL2442 | Protease | 0.111501865 | 0 / 1 |
| Carbonic anhydrase III | CA3 | P07451 | CHEMBL2885 | Lyase | 0.111501865 | 0 / 2 |
| Neuronal acetylcholine receptor protein alpha-7 subunit | CHRNA7 | P36544 | CHEMBL2492 | Ligand-gated ion channel | 0.111501865 | 10 / 0 |
| Telomerase reverse transcriptase | TERT | O14746 | CHEMBL2916 | Enzyme | 0.111501865 | 5 / 3 |
| Carbonic anhydrase II | CA2 | P00918 | CHEMBL205 | Lyase | 0.111501865 | 73 / 4 |
| Carbonic anhydrase I | CA1 | P00915 | CHEMBL261 | Lyase | 0.111501865 | 67 / 3 |
| Carbonic anhydrase VI | CA6 | P23280 | CHEMBL3025 | Lyase | 0.111501865 | 4 / 2 |
| Carbonic anhydrase VA | CA5A | P35218 | CHEMBL4789 | Lyase | 0.111501865 | 5 / 3 |
| DNA polymerase beta (by homology) | POLB | P06746 | CHEMBL2392 | Enzyme | 0.111501865 | 0 / 3 |
| Plasminogen activator inhibitor-1 | SERPINE1 | P05121 | CHEMBL3475 | Secreted protein | 0.111501865 | 2 / 4 |
| Beta amyloid A4 protein | APP | P05067 | CHEMBL2487 | Membrane receptor | 0.111501865 | 13 / 3 |
| Phospholipase A2 group V | PLA2G5 | P39877 | CHEMBL4323 | Enzyme | 0.111501865 | 0 / 3 |
| Group X secretory phospholipase A2 | PLA2G10 | O15496 | CHEMBL4342 | Enzyme | 0.111501865 | 0 / 1 |
| Plasminogen | PLG | P00747 | CHEMBL1801 | Protease | 0.111501865 | 2 / 0 |
| NADPH oxidase 4 | NOX4 | Q9NPH5 | CHEMBL1250375 | Enzyme | 0.111501865 | 8 / 0 |
| Carbonic anhydrase XIII | CA13 | Q8N1Q1 | CHEMBL3912 | Lyase | 0.111501865 | 2 / 2 |
| Carbonic anhydrase VB | CA5B | Q9Y2D0 | CHEMBL3969 | Lyase | 0.111501865 | 3 / 2 |
| Acetylcholinesterase | ACHE | P22303 | CHEMBL220 | Hydrolase | 0.111501865 | 10 / 50 |
| Beta-glucuronidase | GUSB | P08236 | CHEMBL2728 | Enzyme | 0.111501865 | 8 / 0 |
| Acyl coenzyme A:cholesterol acyltransferase | CES1 | P23141 | CHEMBL2265 | Enzyme | 0.111501865 | 2 / 1 |
| Sodium/glucose cotransporter 2 | SLC5A2 | P31639 | CHEMBL3884 | Electrochemical transporter | 0.111501865 | 2 / 3 |
| Carboxylesterase 2 | CES2 | O00748 | CHEMBL3180 | Enzyme | 0.111501865 | 1 / 1 |
| Retinoid X receptor alpha | RXRA | P19793 | CHEMBL2061 | Nuclear receptor | 0.111501865 | 0 / 18 |
| Vascular endothelial growth factor receptor 2 | KDR | P35968 | CHEMBL279 | Kinase | 0.111501865 | 63 / 12 |
| Metabotropic glutamate receptor 2 (by homology) | GRM2 | Q14416 | CHEMBL5137 | Family C G protein-coupled receptor | 0.111501865 | 0 / 72 |
| Aldose reductase | AKR1B1 | P15121 | CHEMBL1900 | Enzyme | 0.111501865 | 59 / 43 |
| Cyclin-dependent kinase 1/cyclin B1 | CDK1 CCNB1 | P06493 P14635 | CHEMBL1907602 | Other cytosolic protein | 0.111501865 | 5 / 0 |
| Cyclin-dependent kinase 2/cyclin E1 | CCNE1 CDK2 | P24864 P24941 | CHEMBL1907605 | Kinase | 0.111501865 | 10 / 0 |
| Dual specificity protein phosphatase 3 | DUSP3 | P51452 | CHEMBL2635 | Phosphatase | 0.111501865 | 3 / 0 |
| Nerve growth factor receptor Trk-A | NTRK1 | P04629 | CHEMBL2815 | Kinase | 0.111501865 | 3 / 0 |
| Serine/threonine-protein kinase Aurora-A | AURKA | O14965 | CHEMBL4722 | Kinase | 0.111501865 | 33 / 0 |
| Carbonic anhydrase IX | CA9 | Q16790 | CHEMBL3594 | Lyase | 0.111501865 | 53 / 1 |
| DNA topoisomerase I | TOP1 | P11387 | CHEMBL1781 | Isomerase | 0.111501865 | 4 / 0 |
| DNA (cytosine-5)-methyltransferase 1 | DNMT1 | P26358 | CHEMBL1993 | Writer | 0.111501865 | 0 / 1 |
| 6-phosphogluconate dehydrogenase | PGD | P52209 | CHEMBL3404 | Enzyme | 0.111501865 | 0 / 4 |
| CMP-N-acetylneuraminate-beta-1,4-galactoside alpha-2,3-sialyltransferase | ST3GAL3 | Q11203 | CHEMBL3596076 | Transferase | 0.111501865 | 0 / 1 |
| Alpha-(1,3)-fucosyltransferase 7 | FUT7 | Q11130 | CHEMBL3596077 | Transferase | 0.111501865 | 0 / 1 |
| Fucosyltransferase 4 | FUT4 | P22083 | CHEMBL4996 | Enzyme | 0.111501865 | 0 / 1 |
| Signal transducer and activator of transcription 1-alpha/beta | STAT1 | P42224 | CHEMBL6101 | Transcription factor | 0.111501865 | 0 / 1 |
| Squalene monooxygenase (by homology) | SQLE | Q14534 | CHEMBL3592 | Enzyme | 0.111501865 | 0 / 3 |
| Free fatty acid receptor 1 | FFAR1 | O14842 | CHEMBL4422 | Family A G protein-coupled receptor | 0.111501865 | 0 / 129 |
| Serine/threonine-protein kinase PIM1 | PIM1 | P11309 | CHEMBL2147 | Kinase | 0.111501865 | 12 / 0 |
| Serine/threonine-protein kinase PIM2 | PIM2 | Q9P1W9 | CHEMBL4523 | Kinase | 0.111501865 | 6 / 0 |
| Serine/threonine-protein kinase PIM3 | PIM3 | Q86V86 | CHEMBL5407 | Kinase | 0.111501865 | 3 / 0 |
| Matrix metalloproteinase 2 | MMP2 | P08253 | CHEMBL333 | Protease | 0.111501865 | 145 / 8 |
| Insulin-like growth factor I receptor | IGF1R | P08069 | CHEMBL1957 | Kinase | 0.111501865 | 15 / 0 |
| Matrix metalloproteinase 9 | MMP9 | P14780 | CHEMBL321 | Protease | 0.111501865 | 167 / 1 |
| Ornithine decarboxylase | ODC1 | P11926 | CHEMBL1869 | Lyase | 0.111501865 | 0 / 5 |
| Poly [ADP-ribose] polymerase-1 | PARP1 | P09874 | CHEMBL3105 | Enzyme | 0.111501865 | 20 / 0 |
| Dynamin-1 | DNM1 | Q05193 | CHEMBL4958 | Enzyme | 0.111501865 | 6 / 0 |
| Tankyrase-2 | TNKS2 | Q9H2K2 | CHEMBL6154 | Enzyme | 0.111501865 | 4 / 0 |
| Tankyrase-1 | TNKS | O95271 | CHEMBL6164 | Enzyme | 0.111501865 | 4 / 0 |
| Hepatocyte growth factor receptor | MET | P08581 | CHEMBL3717 | Kinase | 0.111501865 | 56 / 5 |
| Dual-specificity tyrosine-phosphorylation regulated kinase 1A | DYRK1A | Q13627 | CHEMBL2292 | Kinase | 0.111501865 | 23 / 1 |
| P-glycoprotein 1 | ABCB1 | P08183 | CHEMBL4302 | Primary active transporter | 0.111501865 | 1 / 188 |
| Peroxisome proliferator-activated receptor gamma | PPARG | P37231 | CHEMBL235 | Nuclear receptor | 0.111501865 | 1 / 118 |
| Mitogen-activated protein kinase kinase kinase kinase 4 | MAP4K4 | O95819 | CHEMBL6166 | Kinase | 0.111501865 | 3 / 0 |
| Phospholipase A2 group IIA | PLA2G2A | P14555 | CHEMBL3474 | Enzyme | 0.111501865 | 1 / 8 |
| Cyclin-dependent kinase 2/cyclin A | CDK2 CCNA1 CCNA2 | P24941 P78396 P20248 | CHEMBL2094128 | Other cytosolic protein | 0.111501865 | 13 / 0 |
| Protein-arginine N-methyltransferase 1 | PRMT1 | Q99873 | CHEMBL5524 | Writer | 0.111501865 | 4 / 0 |
| Estradiol 17-beta-dehydrogenase 2 | HSD17B2 | P37059 | CHEMBL2789 | Enzyme | 0.111501865 | 44 / 0 |
| Endothelin receptor ET-A | EDNRA | P25101 | CHEMBL252 | Family A G protein-coupled receptor | 0.111501865 | 12 / 81 |
| Apoptosis regulator Bcl-2 | BCL2 | P10415 | CHEMBL4860 | Other ion channel | 0.111501865 | 6 / 10 |
| Serine/threonine-protein kinase/endoribonuclease IRE1 | ERN1 | O75460 | CHEMBL1163101 | Enzyme | 0 | 22 / 0 |
| Estradiol 17-beta-dehydrogenase 3 | HSD17B3 | P37058 | CHEMBL4234 | Enzyme | 0 | 5 / 0 |
| Serine/threonine-protein kinase Chk1 | CHEK1 | O14757 | CHEMBL4630 | Kinase | 0 | 39 / 0 |
| Matrix metalloproteinase 8 | MMP8 | P22894 | CHEMBL4588 | Protease | 0 | 74 / 0 |
| Arachidonate 12-lipoxygenase | ALOX12 | P18054 | CHEMBL3687 | Enzyme | 0 | 0 / 4 |
| Purine nucleoside phosphorylase (by homology) | PNP | P00491 | CHEMBL4338 | Enzyme | 0 | 4 / 0 |
| Platelet-derived growth factor receptor beta | PDGFRB | P09619 | CHEMBL1913 | Kinase | 0 | 8 / 0 |
| Matrix metalloproteinase 3 | MMP3 | P08254 | CHEMBL283 | Protease | 0 | 64 / 0 |
| Stem cell growth factor receptor | KIT | P10721 | CHEMBL1936 | Kinase | 0 | 2 / 3 |
| Potassium-transporting ATPase | ATP4B ATP4A | P51164 P20648 | CHEMBL2095173 | Primary active transporter | 0 | 1 / 0 |
| Serine/threonine-protein kinase RAF | RAF1 | P04049 | CHEMBL1906 | Kinase | 0 | 10 / 0 |
| MAP kinase-activated protein kinase 2 | MAPKAPK2 | P49137 | CHEMBL2208 | Kinase | 0 | 7 / 0 |
